# S2k-Leitlinie: Status Epilepticus im Erwachsenenalter: Leitlinie der Deutschen Gesellschaft für Neurologie

**DOI:** 10.1007/s00115-020-01036-2

**Published:** 2021-03-22

**Authors:** F. Rosenow, J. Weber, Deutsche Gesellschaft für Neurologie (DGN), Österreichische Gesellschaft für Neurologie (ÖGN)

**Affiliations:** 1 Epilepsiezentrum Frankfurt Rhein-Main, Klinik für Neurologie, Universitätsklinikum Frankfurt, Theodor-Stern-Kai 7, 60590 Frankfurt/Main, Deutschland; 2 Klinik für Neurologie, Klinikum Klagenfurt, Feschnigstraße 11, 9020 Klagenfurt am Wörthersee, Österreich

**Keywords:** Status epilepticus, Anfallsserie, Refraktärer Status epilepticus, Intravenöse Antiepileptika, Intubationsnarkose, Diagnosen ICD-10 Ziffern G41.0, G41.1, G41.2, G41.8, G41.9, Status epilepticus, Seizure series, Refractory status epilepticus, Intravenous antiepileptics, Intubation anesthesia, Diagnoses ICD-10 codes G41.0, G41.1, G41.2, G41.8, G41.9

## Abstract

Diese S2k-Leitlinie (LL) zum Status epilepticus (SE) im Erwachsenenalter schreibt die letzte DGN-LL zum SE von 2012 fort. Neue Definitionen und Evidenz wurden bei der Erstellung der LL und des Clinical Pathway berücksichtigt. Jeder epileptische Anfall, der länger als 5 Minuten anhält (oder ≥ 2 Anfälle über einen Zeitraum von mehr als 5 Minuten ohne Wiedererlangen des neurologischen Ausgangsstatus), soll als SE behandelt werden.

In der Diagnostik sollte initial eine CCT oder, wenn möglich, eine MRT erfolgen. Das EEG spielt bei der Diagnosestellung und beim Therapiemonitoring von non-konvulsiven SE und zum Ausschluss bzw. Nachweis psychogener nichtepileptischer Anfälle eine wesentliche Rolle. Der prognostische Einfluss von insbesondere entzündlichen Begleiterkrankungen (z. B. Pneumonie) wurde besser belegt, weshalb entsprechende Laborparameter auch im Verlauf kontrolliert werden sollten und ggf. frühzeitig eine antibiotische Therapie initiiert werden sollte.

Die Therapie erfolgt in 4 Stufen: 1. Initialer SE: Gabe eines ausreichend hoch dosierten Benzodiazepins i. m., i. v. oder i. n.; 2. Benzodiazepin-refraktärer SE: 1. Wahl ist die i.v. Gabe von Levetiracetam oder Valproat; 3. Refraktärer SE (RSE) und 4. Superrefraktärer SE (SRSE): I.v. Propofol oder Midazolam alleine oder in Kombination oder Thiopental in anästhetischen Dosen. Beim fokalen non-konvulsiven RSE kann unter Umständen auf die Einleitung eines therapeutischen Komas verzichtet werden. Bei SRSE sollte die ketogene Diät zum Einsatz kommen. I.v. Ketamin oder inhalatives Isofluran kann erwogen werden. In Einzelfällen kann die elektrokonvulsive Therapie und, bei resektabler epileptogener Zone, ein Epilepsie chirurgischer Eingriff erwogen werden. I.v. Allopregnanolon oder die Hyperthermie sollen nicht eingesetzt werden.

## Abstract (en)

This S2k guideline on diagnosis and treatment of status epilepticus (SE) in adults is based on the last published version from 2021. New definitions and evidence were included in the guideline and the clinical pathway. A seizures lasting longer than 5 minutes (or ≥ 2 seizures over more than 5 mins without intermittend recovery to the preictal neurological state. Initial diagnosis should include a cCT or, if possible, an MRI. The EEG is highly relevant for diagnosis and treatment-monitoring of non-convulsive SE and for the exclusion or diagnosis of psychogenic non-epileptic seizures. As the increasing evidence supports the relevance of inflammatory comorbidities (e.g. pneumonia) related clinical chemistry should be obtained and repeated over the course of a SE treatment, and antibiotic therapy initiated if indicated.

Treatment is applied on four levels: 1. Initial SE: An adequate dose of benzodiazepine is given i.v., i.m., or i.n.; 2. Benzodiazepine-refractory SE: I.v. drugs of 1st choice are levetiracetam or valproate; 3. Refractory SE (RSE) or 4. Super-refractory SE (SRSE): I.v. propofol or midazolam alone or in combination or thiopental in anaesthetic doses are given. In focal non-convulsive RSE the induction of a therapeutic coma depends on the circumstances and is not mandatory. In SRSE the ketogenic diet should be given. I.v. ketamine or inhalative isoflorane can be considered. In selected cased electroconvulsive therapy or, if a resectable epileptogenic zone can be defined epilepsy surgery can be applied. I.v. allopregnanolone or systemic hypothermia should not be used

## Was gibt es Neues?

### Zu Diagnostik, Klassifikation, Epidemiologie und Prognose

- Es liegt eine neue ILAE-Klassifikation des Status epilepticus (SE) vor [134], welche die minimale Dauer des generalisierten konvulsiven SE als ≥5 min und die anderer Statusformen mit ≥10 min definiert. Für den Absencenstatus ist die Minimaldauer fraglich. Die Leitlinienkommission hält an der pragmatischen Definition einer Minimaldauer von 5 min für alle Statusformen aus der DGN-Leitlinie 2012 fest.
- Neue EEG-Kriterien für das Vorliegen eines nonkonvulsiven SE wurden definiert und in einer ersten Studie auch validiert [69, 71].
- Das Konzept des superrefraktären SE und dessen Definition gilt mittlerweile als etabliert [36].
- Es liegen neue epidemiologische und Krankheitskostendaten zu nonrefraktärem, refraktärem und superrefraktärem SE vor [64, 116, 117].
- Der Einfluss von Komorbiditäten (z. B. einer Pneumonie) und von Labormarkern von Entzündung auf das neurologische Outcome und die SE-assoziierte Sterblichkeit wurde durch weitere Studien belegt [108, 123, 129].

### Zur Therapie

- Es wurde gezeigt, dass mit einem Applikator gegebenes intramuskuläres Midazolam (10 mg, bis 40 kg 5 mg) in der Initialtherapie des Status generalisierter konvulsiver Anfälle der i.v. Gabe von 4 mg Lorazepam mindestens gleichwertig ist ([112], EG1B). Das Ergebnis wurde vor allem durch die raschere Applikation des fertig aufgezogenen Midazolams aus einem Applikator begründet. Seit Dezember 2019 wird diese Form der Applikation in Deutschland durch die Firma Desitin Arzneimittel vermarktet.
- Intranasales Midazolam-Spray wurde in den USA für die Therapie von Anfallsclustern zugelassen und ist dort seit November 2019 verfügbar. Derzeit wird nicht erwartet, dass es in Deutschland zugelassen werden wird. Erste Studien zum Einsatz von i.n. Midazolam beim SE liegen vor [60] und eine Metaanalyse spricht dafür, dass unter den nicht intravenösen Midazolamapplikationen die i.n. Gabe nach der i.m. Gabe die wirksamste ist [4].
- Auch unter Einbeziehung von Clonazepam und Diazepam liegen weiterhin keine Einzelstudien vor, die klar für die Überlegenheit eines Benzodiazepins bezüglich der Durchbrechungsrate sprechen. Die höchste Evidenz liegt für intravenöses Lorazepam und für intramuskuläres Midazolam (per Applikator) vor.
- Eine große Registerstudie bestätigt die Rolle von Benzodiazepinen in der Initialtherapie des Status epilepticus. Die höchsten Durchbrechungsraten wurden nach der Gabe einer ausreichend hohen Dosis eines Benzodiazepins beobachtet. Häufigster Fehler war die Gabe zu niedriger Dosen [62].
- In diesem Zusammenhang gibt es Hinweise darauf, dass insbesondere Lorazepam oft zu niedrig dosiert wird (z. B. 2 mg statt 4 mg; [3]).
- Die Initialdosen von Benzodiazepinen bei Erwachsenen bzw. Kindern/Personen mit <40 kgKG liegen bei: Lorazepam **0,1** **mg/kg **(max. 4 mg/Bolusgabe, ggf. 1‑mal wiederholen) oder Clonazepam **0,015** **mg/kg **(max. 1 mg/Bolusgabe, ggf. 1‑mal wiederholen) oder Midazolam **0,2** **mg/kg **(max. 10 mg/Bolusgabe i.m., i.v. oder i.n. [bei <40–13 kgKG 5 mg], ggf. 1‑mal wiederholen) oder Diazepam **0,15–0,2** **mg/kg **(max. 10 mg/Bolusgabe, ggf. 1‑mal wiederholen; [3, 46, 59]).
- Eine komparative Studie zur Stufe 2 spricht dafür, dass in der Therapie des benzodiazepinrefraktären konvulsiven SE Levetiracetam (LEV, 60 mg/kg, max. 4500 mg), Fosphenytoin (FPHT, 20 mg/kg, max. 1500 mg) und Valproat (VPA, 40 mg/kg, max. 3000 mg) von vergleichbarer Effektivität sind. Der primäre Effektivitätsendpunkt (das Sistieren des Status bei Besserung des Bewusstseins) wurde in 47 % (LEV), 45 % (FPHT) und 46 % (VPA) erreicht. Dabei war auch die Verträglichkeit nicht signifikant verschieden [59].
- Zwei kontrollierte Komparatorstudien haben prospektiv die Wirksamkeit und Verträglichkeit von Phenytoin 20 mg/kg und Levetiracetam 40 mg/kg bei Kindern und Jugendlichen im benzodiazepinrefraktären konvulsiven SE verglichen und vergleichbare Wirksamkeit und Verträglichkeit berichtet [25, 76].
- Es liegen mehrere neue retrospektive Kohortenstudien zur Gabe verschiedener intravenös applizierbarer Antiepileptika vor. Diese Studien sprechen in ihrer Summe dafür, dass neben Levetiracetam (nicht zur SE-Therapie zugelassen), Valproat (eingeschränkt zur SE-Therapie zugelassen), Fosphenytoin, Phenytoin und Phenobarbital auch Lacosamid (nicht zur SE-Therapie zugelassen) im Allgemeinen nur vergleichbar geringe Nebenwirkungs- und Komplikationsraten aufweist.
- In einer prospektiven kontrollierten Studie wurden Phenytoin und Lacosamid bezüglich ihrer Wirksamkeit und Verträglichkeit bei Patienten mit nonkonvulsiven Anfällen im EEG-Monitoring verglichen. Es ergaben sich keine signifikanten Unterschiede [56].
- Eine Überlegenheit der gleichzeitigen Gabe von Clonazepam und Levetiracetam gegenüber der alleinigen Gabe von Clonazepam durch den Rettungssanitäter vor Ort konnte nicht gezeigt werden [90].
- Mit Brivaracetam (nicht zur SE-Therapie zugelassen) steht seit 2016 ein weiteres intravenös applizierbares Antiepileptikum zur Verfügung, welches ersten Untersuchungen zufolge rascher als Levetiracetam eine zerebrale Maximalkonzentration erreicht [15, 38]. Erste Anwendungen in der Statustherapie wurden bereits berichtet [105, 115].
- Die therapeutische systemische Hypothermie (32–34 ^°^C über 24 h) konnte in einer randomisierten kontrollierten Studie bei intubierten Patienten die Entwicklung zu einem RSE oder SRSE nicht verhindern, zudem zeigte sich kein signifikanter Effekt auf das neurologische Outcome nach 90 Tagen [68].
- Allopregnanolon i.v. war in einer randomisierten kontrollierten Studie der Gabe von Placebo in der Therapie des superrefraktären SE nicht überlegen (https://investor.sagerx.com […]).
- Ketamin i.v. konnte nach zwei retrospektiven Studien bei Patienten mit SRSE (nach Ausschluss einer posthypoxischen Ursache) jenseits von Propofol den SRSE in 40 % der Fälle durchbrechen [43, 53].

## Die wichtigsten Empfehlungen auf einen Blick

### Empfehlungen zur Diagnostik

1. Definition: Jeder epileptische Anfall, der länger als 5 min anhält (oder ≥2 aufeinanderfolgende Anfälle über einen Zeitraum von mehr als 5 min ohne Wiedererlangen des präiktalen neurologischen Ausgangsstatus), soll als Status epilepticus bezeichnet und behandelt werden (Empfehlungsstärke: stark, Konsensstärke: starker Konsens).
2. Die Klassifikation soll nach ILAE-Vorschlag von 2015 auf der Basis von Semiologie (V. a. motorische Symptome und Grad der Bewusstseinsstörung), Ätiologie, EEG und Alter vorgenommen werden (Empfehlungsstärke: stark, Konsensstärke: starker Konsens).
3. Konvulsive Statusformen sollen in der Regel klinisch diagnostiziert werden (Empfehlungsstärke: stark, Konsensstärke: starker Konsens).
4. Wenn eine Abgrenzung zu psychogenem Status klinisch nicht möglich ist und bei V. a. nonkonvulsive Statusformen soll das EEG eingesetzt werden (Empfehlungsstärke: stark, Konsensstärke: starker Konsens).
5. Bei klinischem Verdacht auf einen nonkonvulsiven SE soll die Sensitivität des EEG durch längere Aufzeichnungszeiten und häufiges Wiederholen erhöht werden (Empfehlungsstärke: stark, Konsensstärke: starker Konsens).
6. In der Akutsituation bei neu diagnostiziertem SE soll zum Ausschluss akuter symptomatischer Ätiologien ein cCT (bei Verfügbarkeit alternativ ein MRT) erfolgen (Empfehlungsstärke: stark, Konsensstärke: starker Konsens).
7. Im Verlauf eines SE soll bei bisher unklarer Ätiologie ein MRT erfolgen (Empfehlungsstärke: stark, Konsensstärke: starker Konsens).
8. Laboruntersuchungen zu Beginn eines SE sollen die folgenden Standardparameter im Serum beinhalten: Blutbild, Elektrolyte, Leberfunktions- und Nierenretentionswerte, CK, Glukose, Schilddrüsenhormone, toxikologische Untersuchung (ggf. auch im Urin). Bei Fieber sollen die Entzündungsparameter sowie mikrobiologische Untersuchungen erfolgen (ggf. auch im Liquor). Bei bekannter Epilepsie sollen die Serumkonzentrationen der aktuell verschriebenen Antiepileptika bestimmt werden (Empfehlungsstärke: stark, Konsensstärke: starker Konsens).
9. Bei klinischem Verdacht sollen rheumatologische Erkrankungen, Autoimmunprozesse mit antineuronalen Antikörpern und angeborene chromosomale, mitochondriale und metabolische Erkrankungen nachgewiesen bzw. ausgeschlossen werden (Empfehlungsstärke: stark, Konsensstärke: Konsens).
10. Im Krankheitsverlauf sollten regelmäßig (initial mindestens täglich) die folgenden Parameter bestimmt werden: Blutbild, Entzündungsparameter, Elektrolyte, Nieren- und Leberwerte, CK, Lactat, Blutgasanalyse und Medikamentenspiegel (bei Valproatgabe auch die freie Fraktion) (Empfehlungsstärke: Empfehlung, Konsensstärke: starker Konsens).
11. Differenzialdiagnostisch sollen die folgenden Erkrankungen erwogen und ggf. ausgeschlossen werden: Status psychogener Anfälle, hypoxische Enzephalopathie, toxische oder metabolische Enzephalopathie und Tetanus (Empfehlungsstärke: stark, Konsensstärke: starker Konsens).

### Empfehlungen zur Therapie und Versorgungskoordination

#### Stufe 1 (Therapie des initialen Status epilepticus einschließlich der Prähospitalphase)

12. Die Sicherstellung der Vitalparameter (ABCDE-Schema) und der Schutz vor Selbstgefährdung sollen gewährleistet werden (Empfehlungsstärke: stark, Konsensstärke: starker Konsens).
13. Da eine Intubationsbereitschaft immer gesichert sein muss, sollte im Zielkrankenhaus eine Intensivüberwachung erfolgen (Empfehlungsstärke: Empfehlung, Konsensstärke: Konsens).
14. Bei etabliertem intravenösem Zugang soll bei Erwachsenen bzw. Kindern/Personen mit >13 kgKG gegeben werden: Lorazepam 0,1 mg/kg (max. 4 mg/Bolusgabe, ggf. 1‑mal wiederholen) oder Clonazepam 0,015 mg/kg (max. 1 mg/Bolusgabe, ggf. 1‑mal wiederholen) oder Midazolam 0,2 mg/kg (max. 10 mg/Bolusgabe [<40–13 kgKG 5 mg], ggf. 1‑mal wiederholen) oder Diazepam 0,15–0,2 mg/kg (max. 10 mg/Bolusgabe, ggf. 1‑mal wiederholen). Bei Patienten ohne i.v.-Zugang sollte Midazolam intramuskulär per Applikator oder intranasal (10 mg für >40 kg, 5 mg für <40–13 kgKG als Einzelgabe) appliziert werden (Empfehlungsstärke: stark, Konsensstärke: starker Konsens).
15. Diazepam rektal (0,2–0,5 mg/kg, max. 20 mg/Gabe, Einzelgabe) oder Midazolam bukkal können bei fehlendem i.v. Zugang alternativ zu Midazolam i.n. oder i.m. angewendet werden (Empfehlungsstärke: offen, Konsensstärke: starker Konsens).

#### Empfehlungen zu Stufe 2 (Therapie des benzodiazepinrefraktären Status epilepticus)

16. Bei Persistenz des SE nach der initialen Gabe eines Benzodiazepins soll geprüft werden, ob die Dosis adäquat war, denn Unterdosierungen sind häufig und mit verminderten Kontrollraten assoziiert. Ggf. soll erneut ein Benzodiazepin appliziert werden (Empfehlungsstärke: stark, Konsensstärke: starker Konsens).
17. Wenn die initiale Benzodiazepingabe ausreichend hoch dosiert war (und weniger als 30 min zurückliegt) sollen in der 2. Therapiestufe i.v. verfügbare Antiepileptika (AED) gegeben werden (Empfehlungsstärke: stark, Konsensstärke: starker Konsens).
18. Als Medikamente der 1. Wahl sollen Levetiracetam (LEV, 60 mg/kg, max. 4500 mg über >10 min), Valproat (VPA, 40 mg/kg, max. 3000 mg über >10 min) oder Fosphenytoin (FPHT, 20 mg/kg, max. 1500 mg über >10 min) gegeben werden (Empfehlungsstärke: stark, Konsensstärke: starker Konsens).
  (Anmerkungen zum Zulassungsstatus: LEV ist in Europa aktuell nicht zur Therapie des SE zugelassen. VPA ist beim Absencenstatus als 1. Wahl, beim fokalen nonkonvulsiven SE als 2. Wahl nach Benzodiazepinen und beim konvulsiven SE als Mittel der 3. Wahl zugelassen. FPHT ist zwar in Deutschland und Österreich zugelassen, wird aber nicht vermarktet und ist in der Schweiz nicht zugelassen.)
19. Zugelassen zur Therapie des SE sind auch Phenytoin und Phenobarbital und sollen alternativ eingesetzt werden, insbesondere, wenn bei vorbestehender Epilepsie subtherapeutische Spiegel einer dieser Substanzen festgestellt wurden (Empfehlungsstärke: stark, Konsensstärke: starker Konsens).
20. Auch i.v. Lacosamid sollte als Medikament der 2. Wahl eingesetzt werden (Initialdosis 5 mg/kg über 15–30 min; z. T. wird 200 mg/15 min als maximale Infusionsgeschwindigkeit vorgeschlagen); (Empfehlungsstärke: Empfehlung, Konsensstärke: Konsens).
  (Anmerkung zum Zulassungsstatus: Lacosamid ist nicht zur Therapie des Status epilepticus zugelassen.)

#### Empfehlungen zu Stufe 3 und 4 (Therapie des refraktären und superrefraktären Status epilepticus)

21. Der refraktäre konvulsive Status epilepticus soll mit Propofol oder Midazolam oder einer Kombination der beiden oder mit Thiopental in anästhetischen Dosen behandelt werden (Empfehlungsstärke: stark, Konsensstärke: starker Konsens).
22. Die Therapie des refraktären konvulsiven Status epilepticus soll auf einer Intensivstation bei intubiertem Patienten erfolgen (Empfehlungsstärke: stark, Konsensstärke: Konsens).
23. Die Therapieeinleitung, deren Überwachung und das Ausschleichen der Anästhetika sollten mittels kontinuierlichen EEG-Monitorings erfolgen (Empfehlungsstärke: Empfehlung, Konsensstärke: Konsens).
24. Beim fokalen nonkonvulsiven refraktären Status epilepticus kann unter Berücksichtigung der klinischen Konstellation auf die Einleitung eines therapeutischen Komas verzichtet werden (Empfehlungsstärke: offen, Konsensstärke: starker Konsens).
25. Bei Patienten mit SRSE sollte die ketogene Diät zum Einsatz kommen (Empfehlungsstärke: Empfehlung, Konsensstärke: Konsens).
26. Bei Patienten mit SRSE kann der Einsatz von hochdosierten Barbituraten (Thiopental) unter Abwägung von Nutzen und Risiken erwogen werden (Empfehlungsstärke: offen, Konsensstärke: starker Konsens).
27. Bei Patienten mit SRSE kann der Einsatz von intravenös appliziertem Ketamin oder inhalativem Isofluran erwogen werden (Empfehlungsstärke: offen, Konsensstärke: starker Konsens).
28. Bei Patienten mit SRSE kann bei identifizierter resektabler epileptogener Zone die resektive Epilepsiechirurgie erwogen werden (Empfehlungsstärke: offen, Konsensstärke: starker Konsens).
29. Bei Patienten mit SRSE kann die elektrokonvulsive Therapie in Einzelfällen erwogen werden (Empfehlungsstärke: offen, Konsensstärke: starker Konsens).
30. Bei Patienten mit SRSE soll Allopregnanolon, nach einem negativen Ergebnis einer randomisierten kontrollierten Studie, nicht eingesetzt werden (Empfehlungsstärke: stark, Konsensstärke: starker Konsens).
31. Bei Patienten mit SRSE soll die systemische Hypothermie mit dem Therapieziel Anfallsunterbrechung und Besserung der funktionellen Prognose auf Basis der aktuellen Datenlage nicht zum Einsatz kommen (Empfehlungsstärke: stark, Konsensstärke: starker Konsens).
32. Bei Einleitung bzw. Fortführung oder Eskalation einer intensivmedizinischen Therapie bei Patienten mit RSE und SRSE sollten der Patientenwille und das Vorliegen einer Patientenverfügung beachtet werden (Empfehlungsstärke: Empfehlung, Konsensstärke: starker Konsens).

## 1 Einführung: Geltungsbereich und Zweck der Leitlinie

### Begründung und Notwendigkeit einer Leitlinie

Weiterentwicklung der bestehenden Leitlinie „Status epilepticus im Erwachsenenalter“ von 2012.

### Ziele der Leitlinie

Verbesserung und Vereinheitlichung der Behandlung von verschiedenen Formen des Status epilepticus im Erwachsenenalter.

### Patientenzielgruppe

Erwachsene Patienten mit einem Status epilepticus.

### Versorgungsbereich

Ambulante Notfalltherapie, Notaufnahmen und stationäre Versorgung in den Bereichen Diagnostik, Therapie und Intensivtherapie.

### Adressaten der Leitlinie

Ärzte folgender Fachrichtungen, die in unterschiedlichen klinischen Kontexten mit der Notfall‑, Erst- und/oder Folgeversorgung von erwachsenen Patienten mit Status epilepticus befasst sind: Neurologen, Neurointensiv- und Notfallmediziner, Epileptologen, Neurophysiologen. Die Leitlinie soll auch als Grundlage für die Beratung und Information von Laienhelfern (z. B. Angehörige oder Pflegende von Patienten mit rezidivierendem Status) durch diese Ärztegruppen dienen.

## 2 Definition, Epidemiologie und Klassifikation

### Definition

Ein Status epilepticus ist ein prolongierter epileptischer Anfall, jede semiologische Form eines epileptischen Anfalls kann zu einem Status epilepticus werden. Ein epileptischer Anfall ist definiert als „ein vorübergehendes Auftreten von (subjektiven) Zeichen und/oder (objektivierbaren) Symptomen auf der Basis anomal exzessiver und/oder synchronisierter neuronaler Aktivität im Gehirn“ [39]. Epileptische Anfälle sind selbstlimitierende Ereignisse, der zugrunde liegende Mechanismus des spontanen Sistierens ist weitgehend unklar. Eine Video-EEG-Untersuchung konnte zeigen, dass die mediane Dauer eines fokalen zu bilateral tonisch-klonischen Anfalls 130 s (Spanne 37–139) und die eines fokalen, nicht bewusst erlebten Anfalls 78 s (Spanne 8–298) beträgt [30, 58, 133].

In der Vergangenheit wurden auf Basis unterschiedlicher konzeptueller Überlegungen Zeitpunkte definiert, ab denen ein epileptischer Anfall zu einem Status epilepticus wird. Tierexperimentelle Untersuchungen an Pavianen aus den 1970er-Jahren konnten zeigen, dass bei länger anhaltender epileptischer Aktivität aufgrund der starken Exzitotoxizität irreversible neuronale Schäden auftreten. Einem konzeptuellen grundlagenwissenschaftlichen Ansatz folgend wurde ein epileptischer Anfall daher ab 30 min Dauer als Status epilepticus definiert [32]. Dieser Zeitpunkt erscheint für epidemiologische Studien durchaus sinnvoll und wird dafür auch heute noch zugrunde gelegt, damit nicht zu viele selbstlimitierende epileptische Anfälle fälschlicherweise als Status epilepticus erfasst werden. Aus rein pragmatischer Sicht ist die Dauer von 30 min jedoch nicht hilfreich, da eine akute pharmakologische Intervention viel früher erfolgen muss, um das Risiko für Morbidität und Letalität zu reduzieren [27]. Daher wurde Ende der 1990er-Jahre, einem operationalen klinischen Ansatz folgend, die Definition eines Status epilepticus ab einer Anfallsdauer von 5 min vorgeschlagen [75]. Ergänzt wurde, dass auch ≥2 aufeinanderfolgende Anfälle über einen Zeitraum von mehr als 5 min ohne Wiedererlangen des präiktalen neurologischen Ausgangsstatus einem Status epilepticus entsprechen. Alle bisher genannten Definitionen bezogen sich ausschließlich auf den Status fokal zu bilateral oder generalisierter tonisch-klonischer Anfälle, welcher auch als generalisierter konvulsiver Status epilepticus bezeichnet wird.

Die aktuelle Definition des Status epilepticus der Internationalen Liga gegen Epilepsie aus dem Jahr 2015 berücksichtigt sowohl konzeptuell-grundlagenwissenschaftliche Erkenntnisse als auch klinisch-pragmatische Erfordernisse; zudem werden jenseits des generalisierten konvulsiven Status epilepticus erstmals weitere häufige klinische Formen aufgegriffen [134].

Ab Zeitpunkt T1 ist ein epileptischer Anfall „anomal prolongiert“, ein Überschreiten dieses Zeitpunkts stellt in der Regel die Indikation für die akute Gabe eines anfallsunterbrechenden Medikaments dar. Ab Zeitpunkt T2 besteht die Gefahr, dass irreversible neuronale Schädigungen auftreten können. Einigermaßen belastbare Daten für die Definition von T1 und T2 liegen nur für den generalisierten konvulsiven Status epilepticus vor, für die anderen klinischen Formen können diese nur geschätzt werden (Tab. 1).

**Table 1:** 

Form des SE	T1 Prolongierter Anfall	T2 Risiko bleibender neuronaler Schäden
Generalisiert konvulsiv	5 min	30 min
Fokal, nicht bewusst erlebt	10 min	>60 min
Absence	10–15 min	Unbekannt

*SE* Status epilepticus

Viele Zentren halten jedoch im klinischen Alltag an der älteren Definition fest, dass jeder epileptische Anfall, der länger als 5 min dauert, ein Status epilepticus ist. Dieser pragmatische Ansatz gibt vor allem Nichtepileptologen und Nichtneurologen, die nicht mit allen semiologischen Feinheiten epileptischer Anfälle vertraut sind, Sicherheit beim therapeutischen Management der Patienten.

#### Empfehlung

Jeder epileptische Anfall, der länger als 5 min anhält, definiert einen Status epilepticus und soll akut antiepileptisch behandelt werden (Empfehlungsstärke: stark).

In Abhängigkeit des Nichtansprechens auf die verschiedenen Stufen der pharmakologischen Therapie werden der refraktäre und der superrefraktäre Status epilepticus abgegrenzt. Ersterer liegt vor, wenn der Status epilepticus klinisch oder rein elektroenzephalographisch anhält, nachdem ein Benzodiazepin und ein klassisches Antiepileptikum in ausreichend hoher Dosis gegeben worden ist. Man spricht von einem superrefraktären Status epilepticus, wenn die klinische und/oder elektroenzephalographische Anfallsaktivität nach Einleitung einer Therapie mit kontinuierlich applizierten Anästhetika anhält oder zeitnah nach initial erfolgreicher Therapie wieder auftritt, dies beinhaltet auch die Reduktion und/oder das Absetzen der Anästhetika [109].

### Epidemiologie

Der Status epilepticus hat in Europa eine Inzidenz von 10–30/100.000 Einwohner. Die Sterblichkeit der konvulsiven SE liegt bei Erwachsenen bei durchschnittlich 15,9 % (12,7–19,2) und ist vor allem von Alter und Ätiologie abhängig. Sie ist am niedrigsten bei Kindern und am höchsten bei refraktären SE mit lebensbedrohlicher Ätiologie [63, 72, 73, 91].

### Klassifikation

Die aktuelle Klassifikation des Status epilepticus der Internationalen Liga gegen Epilepsie hat das Ziel, belastbare und reproduzierbare Rahmenbedingungen für die klinische Diagnose, für notwendige Untersuchungen sowie für therapeutische Herangehensweisen zu schaffen. Dafür wurden vier Achsen implementiert: Semiologie, Ätiologie, EEG-Korrelate und Alter [134]. Idealerweise sollte jeder Patient mit Status epilepticus entlang dieser vier Achsen kategorisiert werden; dies wird aber im klinischen Alltag häufig nicht möglich sein.

#### Semiologie

Die beiden maßgeblichen semiologischen Kriterien sind:

- An- und Abwesenheit von prominenten motorischen Zeichen
- Vorhandensein und ggf. Ausmaß einer qualitativen und quantitativen Bewusstseinsstörung

Klinische Formen mit relevanten motorischen Zeichen werden als – fokaler oder generalisierter – konvulsiver Status epilepticus bezeichnet; eine Bewusstseinsstörung findet sich bei der generalisierten konvulsiven Form obligat und bei der fokalen Form fakultativ. Finden sich keine relevanten motorischen Zeichen, spricht man von einem nonkonvulsiven Status epilepticus, hier gibt es fokale und generalisierte Formen, zudem kann das Bewusstsein eingeschränkt sein oder nicht.

Die Internationale Liga gegen Epilepsie hat 2017 eine neue Klassifikation sowie eine neue Terminologie von epileptischen Anfällen eingeführt [40]. Diese ist oftmals nicht kongruent mit der Terminologie der verschiedenen Formen des Status epilepticus von 2015. Ein pragmatischer Ansatz wäre, dass man von einem Status von Anfällen einer spezifischen Semiologie spricht, z. B. Status bilateral tonisch-klonischer Anfälle, Status fokaler, nicht bewusst erlebter Anfälle oder Status von Absencen. Letztlich werden aber im klinischen Alltag – wie in der Epileptologie insgesamt – weiterhin seit Langem gebräuchliche Begriffe auch für die verschiedenen Formen des Status epilepticus verwendet, die den offiziellen Termini mitunter nicht entsprechen.

#### Ätiologie

Analog zur ätiologischen Stratifizierung von Epilepsien [106] greift man auch beim Status epilepticus in einem ersten Schritt auf die Kategorien „strukturell“, „metabolisch“, „infektiös“, „immunvermittelt“ sowie „unbekannt“ zurück. Danach erfolgt ggf. eine weitere Spezifizierung, wie „zerebrale Ischämie“ oder „Herpes-Enzephalitis“. Die bei Epilepsien vorhandene ätiologische Kategorie „genetisch“ lässt sich auf den Status epilepticus nicht übertragen. Im Rahmen von generalisierten genetischen Epilepsien kann es zwar zu einem Status epilepticus kommen, die akute Ursache ist dann aber z. B. ein Abfall der Serumkonzentration von Antiepileptika, Fieber oder die zusätzliche Einnahme von prokonvulsiven Medikamenten.

Basierend auf dem zeitlichen Zusammenhang zwischen Auftreten der Ursache und Manifestation des Status epilepticus unterteilt man in akut-symptomatische, unprovozierte (englisch „remote“ für „zeitlich entfernt liegend“) und progressiv verlaufende Ursachen.

#### EEG-Korrelate

Viele Status epileptici gehen mit typischen EEG-Mustern einher. Allerdings gibt es periodische EEG-Muster (LPD), die sowohl im Status epilepticus als auch bei akuten Läsionen wie z. B. bei der Herpes-Enzephalitis oder Schlaganfällen ohne epileptische Anfälle auftreten. Generalisierte periodische Muster im Status epilepticus müssen von EEG-Mustern bei Enzephalopathien abgegrenzt werden [12]. Zur besseren Klassifikation und Systematik des EEG im Status epilepticus gibt es Vorschläge [71] und Empfehlungen der American Clinical Neurophysiology Society [52], die demnächst kommentiert in deutscher Übersetzung in der Zeitschrift Klinische Neurophysiologie erscheinen werden.

Letztlich basiert die Diagnose eines Status epilepticus auf dem Zusammenspiel von Anamnese, klinischen Befunden und iktalem EEG.

Gerade bei Patienten im Koma ist die diagnostische Abgrenzung eines nonkonvulsiven Status epilepticus, basierend auf dem EEG, eine besondere Herausforderung. Die „Salzburg-Kriterien“ schlagen in dieser Konstellation folgende Bedingungen für die Diagnose eines nonkonvulsiven Status epilepticus vor, die Erfüllung einer Bedingung ist ausreichend [47, 69, 71]):

- Frequenz der periodischen Entladungen >2,5/s
- Typische räumlich-zeitliche Ausbreitung der periodischen Aktivität
- Zeitliche Assoziation der periodischen Aktivität mit subtilen klinischen Phänomenen
- Klinisches und elektroenzephalographisches Ansprechen auf intravenös applizierte Antiepileptika (cave: auch die periodischen Entladungen bei nicht epileptischen Enzephalopathien sistieren mit der Gabe von z. B. Benzodiazepinen, der klinische Zustand des Patienten ändert sich aber nicht)

#### Alter

Bestimmte Formen des Status epilepticus manifestieren sich bevorzugt in spezifischen – insbesondere pädiatrischen – Altersgruppen. Ein Status myoclonicus im Rahmen einer bekannten juvenilen myoklonischen Epilepsie, welcher z. B. durch Trigger wie Schlafentzug oder die Einnahme von bei diesem Epilepsiesyndrom ungeeigneten Antiepileptika wie Carbamazepin/Oxcarbazepin oder Gabapentin ausgelöst wird, findet sich gehäuft im Jugendalter und bei jungen Erwachsenen.

Daher sollte bei der Kategorisierung von Patienten mit Status epilepticus die jeweilige Altersgruppe Berücksichtigung finden. Die Internationale Liga gegen Epilepsie schlägt für diese Achse die Einteilung in folgende Altersgruppen vor:

- Neonaten (0–30 Tage)
- Kleinkinder (>1 Monat bis 2 Jahre)
- Kindheit (>2 bis 12 Jahre)
- Jugend und Erwachsenenalter (>12 bis 59 Jahre)
- Höheres Lebensalter (>60 Jahre)

## 3 Diagnostik

### 3.1 Klinische Diagnostik

Der generalisierte konvulsive SE ist klinisch leicht zu diagnostizieren, wenn er in seiner kontinuierlichen Form auftritt. Wenn bilateral konvulsive bzw. generalisiert tonisch-klonische Anfälle intermittierend auftreten, ohne dass zwischenzeitlich der Bewusstseinszustand vor Auftreten des SE wiedererlangt wird, besteht die Schwierigkeit, dass oft der Ausgangsbefund nicht bekannt ist. Hier müssen Annahmen bzw. die Fremdanamnese herangezogen werden. Die relevanteste DD ist der psychogene nicht epileptische Status. Auch dieser lässt sich oft klinisch anhand der Anfallssymptome gut diagnostizieren (s. unten). Wenn klinisch keine eindeutige Abgrenzung gelingt, sind Fremdanamnese/Vorgeschichte, EEG, Labor und gelegentlich auch die Bildgebung hilfreich [54].

Beim nonkonvulsiven SE ist es sehr wichtig, überhaupt einen klinischen Verdacht zu haben. Häufig finden sich neben der Bewusstseinsalteration geringe, leicht zu übersehende klinische Symptome wie myoklonische Entäußerungen der M. orbicularis oculi oder distaler Extremitätenmuskeln oder Verhaltensauffälligkeiten, die fluktuieren können [79]. Beim nonkonvulsiven SE ist das Vorliegen eines Komas ein ungünstiger Prädiktor für das klinische Outcome und sollte dokumentiert werden [131]. Bei klinischer Unsicherheit sind Fremdanamnese/Vorgeschichte, EEG, Labor, Liquoranalytik und gelegentlich auch die Bildgebung hilfreich. Ein EEG sollte durchgeführt werden.

Wenn die diagnostischen Möglichkeiten einer Einrichtung erschöpft sind und keine differenzialdiagnostische Klärung möglich ist, sollte die Verlegung in ein Zentrum mit Expertise und Verfügbarkeit eines EEG-Monitorings erwogen werden.

### 3.2 EEG-Diagnostik

Die Elektroenzephalographie (EEG) ist die spezifische technische Untersuchungsmethode für Epilepsien. Sie wird beim Status epilepticus zur Primärdiagnostik und zur Therapiekontrolle eingesetzt. In der Primärdiagnostik des konvulsiven Status epilepticus ist sie zur Abgrenzung von psychogenen Anfällen relevant. Ein Status generalisierter tonisch-klonischer Anfälle wird klinisch diagnostiziert. In der Primärdiagnostik des nicht konvulsiven Status hat die EEG-Diagnostik entscheidende Bedeutung. In der Therapiekontrolle wird das EEG vor allem beim nicht konvulsiven Status angewendet.

Die Sensitivität des EEG hängt von der Aufzeichnungsdauer ab. Dem einzelnen EEG kann ein Anfallsmuster entgehen, vor allem beim länger dauernden Status epilepticus können eindeutige Anfallsmuster seltener auftreten. Daher sollte bei ausreichendem klinischem Verdacht die Sensitivität durch längere Aufzeichnungsdauer bzw. häufiges Wiederholen erhöht werden [33, 41, 120].

Die Spezifität der EEG für epilepsietypische Muster ist durch einige Muster eingeschränkt, die spitz und rhythmisch imponieren, aber keine Korrelation zu Anfällen oder Status epileptici haben. EEG-Kriterien zur Bewertung des EEG im Status epilepticus helfen in der differenzialdiagnostischen Abgrenzung von Enzephalopathien und liefern auch prognostische Daten [33, 69, 71].

### 3.3 Labordiagnostik

Die Labordiagnostik im Status epilepticus kann einerseits zur Ursachenabklärung beitragen, andererseits kann sie mithelfen, frühzeitig Begleit- und Folgeerkrankungen zu erkennen.

Laboruntersuchungen zu Beginn eines SE umfassen die folgenden Standardparameter im Serum: Blutbild, Elektrolyte (inkl. Magnesium), Leberfunktions- und Nierenretentionswerte, Creatinkinase (CK), Glukose, Schilddrüsenhormone, toxikologische Untersuchung (je nach Verfügbarkeit und Bestimmungsgeschwindigkeit auch im Urin). Bei den toxikologischen Untersuchungen ist insbesondere auf die Spiegel/Nachweis von Alkohol, Kokain und Amphetaminen und künstlichen Derivaten zu achten. Bei Fieber sind die Entzündungsparameter (CRP, allenfalls Procalcitonin [PCT]; [121]) zu bestimmen sowie mikrobiologische Untersuchungen (erregerspezifischer Antikörpernachweis, PCR, Kultur) je nach vermutetem Infektfokus in den jeweiligen Körperflüssigkeiten (Blut, Urin, Liquor) vorzunehmen.

Bei Patienten mit einer bekannten Epilepsie müssen die Serumkonzentrationen der aktuell verschriebenen Antiepileptika gemessen werden.

Beim therapierefraktären SE sollten seltenere Ursachen gesucht werden. Dazu gehört die Bestimmung von rheumatologischen (BSG, Vaskulitisscreening), Anti-Thyroideaperoxidase(TPO)-, Thyreoidea-Rezeptor(TR)-stimulierenden Antikörpern [51], paraneoplastischen Antikörpern (vor allem Anti-Hu, Anti-Ma1/2) und insbesondere antineuronalen Antikörpern (vor allem Anti-GABAA, -GABAB, -GAD, -NMDA und -LGI1; [24, 48, 55, 114]). Bei pädiatrischen Patienten sind zudem angeborene chromosomale, mitochondriale und metabolische Erkrankungen zu suchen.

Hinsichtlich (Früh‑)Erkennung von Begleit- und Folgeerkrankungen oder sich anbahnenden ernsten Medikamentennebenwirkungen im SE ist im Verlauf die regelmäßige (initial mindestens tägliche) Bestimmung von Blutbild, Entzündungsparametern, Elektrolyten, Nieren- und Leberwerten (inkl. Ammoniak, wenn Valproat und/oder Topiramat verabreicht werden), CK, Lactat, Blutgasanalysen und Medikamentenspiegeln wichtig. Bei Valproatgabe ist auch die Bestimmung der freien Fraktion bedeutsam, da diese essenziell vom Albumingehalt sowie von der Gesamtdosis abhängig ist (approximatives Berechnungsschema aus Gesamt-VPA-Spiegel in: Hermida und Tutor [50]).

Da Infekte [108, 123, 124, 141] vor allem auch bei Patienten mit refraktärem SE in der Intensivstation (ITS; [122, 125]) eine negative prognostische Rolle bezüglich der Wahrscheinlichkeit, im Krankenhaus zu versterben, spielen (OR: 5,2 [122]), ist das Monitoring der üblichen Infektparameter (Leukozyten, CRP; [121]), aber auch des sensitiveren Procalcitonins (proCT) wertvoll, welches hilft, zwischen bakteriellen und anderen Infekten bzw. zentralem Fieber zu unterscheiden [127].

Im Tiermodell [113] sowie begrenzt beim Menschen gibt es Hinweise, dass im Labor die neuronenspezifische Enolase (NSE) den neuronalen Zerfall unter dem nicht anoxiebedingten SE widerspiegeln könnte [26].

### 3.4 Bildgebung

In der Akutsituation sollte bei neu aufgetretenem Status epilepticus ein cCT (bei Verfügbarkeit alternativ ein MRT) durchgeführt werden. Bei ca. der Hälfte der Patienten mit Status ist keine Epilepsie bekannt, insbesondere bei diesen Patienten sollte im Verlauf zügig ein MRT erfolgen, um symptomatische und ggf. behandelbare Ursachen zu detektieren. Da die Prognose unter anderem entscheidend von der Ätiologie mit beeinflusst wird, ist die frühe Kenntnis der Ätiologie neben der möglichen Behandlungskonsequenz auch entscheidend zur prognostischen Einschätzung [70].

Im MRT können statusassoziierte Veränderungen vor allem der T2-basierten Sequenzen auftreten und sich Signalsteigerungen, insbesondere des Kortex, in der FLAIR-Wichtung, der T2-Wichtung und der diffusionsgewichteten Bildgebung (gesteigerte DWI und reduzierte ADC) zeigen. Diese sich in der Regel im Verlauf von Wochen nach SE zurückbildenden Veränderungen treten häufig fokal, auch an vom Anfallsursprung entfernten Stellen auf. Zusätzlich finden sich diese Veränderungen auch in subkortikalen Regionen wie Basalganglien, Thalamus (vor allem Pulvinar), Hippocampi und Kleinhirn [44, 82, 83, 85].

Eine weitere diagnostische Herausforderung in der klinischen Notfallsituation ist die Unterscheidung eines SE von akuten Schlaganfällen. Hier gibt es erste Hinweise, dass die Durchführung eines Perfusions-CT in einigen Fällen bei der differenzialdiagnostischen Einordnung helfen kann. Im Anfall kommt es zur regionalen Hyperperfusion, die häufig mehr als ein vaskuläres Territorium betrifft und überwiegend kortikal lokalisiert ist, nach Schlaganfall hingegen zur regionalen, den Gefäßsegmenten folgenden Hypoperfusion. Beim Status epilepticus kann daher, entgegengesetzt zum akuten Schlaganfall, der regionale zerebrale Blutfluss (CF) erhöht bzw. normal und die mittlere Transitzeit (MTT) reduziert bzw. normal sein. Zur Nutzung anderer perfusionsbasierter Techniken wie Arterial-Spin-Labeling (ASL) und Perfusions-MRT gibt es bisher nur vereinzelte Fallberichte, sodass der Einsatz der perfusionsbasierten Messungen momentan keinen Stellenwert in der klinischen Praxis hat und noch weiter untersucht werden sollte [49].

#### Empfehlung

Zusammenfassend soll in der Akutsituation zum Ausschluss akuter symptomatischer Ätiologien ein cCT oder alternativ ein MRT erfolgen. Im Verlauf soll bei weiterhin unklarer Ätiologie ein MRT erfolgen (Empfehlungsstärke: stark).

## 4 Differenzialdiagnosen

Differenzialdiagnostisch sind v. a. Erkrankungen zu unterscheiden, die mit motorischen Entäußerungen und/oder mit einer Vigilanzminderung bis hin zum Koma einhergehen und deswegen mit einem konvulsiven oder nonkonvulsiven Status epilepticus verwechselt werden können.

### 4.1 Status psychogener/dissoziativer Anfälle

Mindestens 10 % der Patienten mit Epilepsie leiden auch unter psychogenen/dissoziativen Anfällen („psychogenic non-epileptic seizure“, PNES; Devinsky et al. [28]). Frauen sind häufiger betroffen als Männer. Die klinische Unterscheidung zu epileptischen Anfällen kann schwierig sein. Es gibt kein Anfallssymptom, das einen PNES sicher und grundsätzlich von einem epileptischen Anfall unterscheidet. Im Rahmen eines PNES können auch Zungenbiss, Enuresis und Zyanose auftreten. Die Dauer nicht epileptischer Anfälle ist aber in der Regel länger als die epileptischer Anfälle [5]. Anfallssymptome, die für PNES sprechen, sind geführte, oft asynchrone Bewegungen der Extremitäten mit wechselnder Intensität und wechselnder Seitenbetonung. Die Modulation der Bewegungen durch Ablenkung oder der Wechsel des Bewegungscharakters durch Schmerzreize kann bei PNES ein weiteres wichtiges Unterscheidungsmerkmal darstellen. Ruckartige und stoßende Beckenbewegungen, sog. „pelvic thrusting“, wurden bei PNES beschrieben, wie auch ein „arc de cercle“. Häufig, aber nicht immer, sind die Augen geschlossen und werden beim Versuch einer Öffnung durch den Untersucher zusammengekniffen. Darüber hinaus fehlen bei PNES typische Zeichen einer epileptischen Semiologie, wie oromandibuläre Automatismen oder tonisch-klonische Bewegungsmuster. Bewusstsein und Reagibilität können während eines PNES wechselnd stark beeinträchtigt sein und undulieren. Patienten mit PNES erinnern sich häufig nicht an die Zeit vor dem Anfall, während dies Patienten mit epileptischen Anfällen gelingt. Epileptische Anfälle können im Gegensatz zu PNES aus dem Schlaf heraus auftreten.

Im EEG eines Status psychogener Anfälle finden sich im Gegensatz zum SE keine epileptischen Anfallsmuster. Somit stellt das EEG die wichtigste apparative Diagnostik zur Unterscheidung beider Erkrankungen dar. Die Schwierigkeit liegt häufig aber in der Interpretation eines EEG, das durch (rhythmische) Muskel- und Bewegungsartefakte stark überlagert ist. Die Bestimmung der Creatinkinase (CK) kann sich bei der Differenzierung als hilfreich erweisen, da sie nach motorischen und insbesondere generalisierten tonisch-klonischen Anfällen erhöht sein kann [89]. Allerdings werden beispielsweise komplexe partielle/dialeptische und automotorische Anfälle durch sie häufig nicht abgebildet und eine Konzentrationserhöhung ist nicht pathognomonisch für epileptische Anfälle. Verletzt sich der Patient während eines PNES, kann auch dies zu einem Anstieg der CK führen. Wichtige Hinweise auf PNES können auch psychiatrische Begleiterkrankungen, vorausgegangener Missbrauch oder Traumata sowie multiple unspezifische somatische Beschwerden geben [5].

### 4.2 Hypoxische Enzephalopathien

Von einem SE abzugrenzen sind enzephalopathische Krankheitsbilder, die metabolischer, septischer oder hypoxischer Genese sein können [7]. Häufig sind posthypoxische Enzephalopathien, die klinisch durch Koma gekennzeichnet sind. Hierbei handelt es sich am ehesten um eine „epileptische Enzephalopathie“, bei der zwar epileptische Charakteristika vorhanden sein können, aber die Enzephalopathie im klinischen Bild und im Hinblick auf Therapie und Prognose ganz im Vordergrund steht [42, 92]. Häufig sind (stimulussensitive) periorale und Extremitätenmyoklonien vorhanden. Im Rahmen der Dezerebration/Dekortikation sowie bei erhöhtem intrakraniellem Druck i.R. einer transtentoriellen und beginnenden transforaminalen Herniation kann es auch zu (stimulussensitiven) Streck- und Beugesynergismen kommen, die mit tonischen Anfällen verwechselt werden können. Im EEG finden sich bei epileptischen (hypoxischen) Enzephalopathien häufig generalisierte, rhythmische oder periodische epilepsietypische Potenziale, wie z. B. Polyspike-Wave-Komplexe oder auch andere generalisierte periodische Potenziale. Diese Komplexe zeigen in der Mehrzahl im EEG keine für SE-Muster typische Frequenzmodulation [98]. Triphasische Wellen lassen sich anhand ihrer Charakteristika unterscheiden [12]. Dazwischen ist das EEG stark verlangsamt oder häufiger vollständig supprimiert, was die schlechte Prognose dieser EEG-Muster untermauert [101]. Da es sich bei diesem EEG-Muster nicht um ein klassisches SE-Muster handelt, sondern vielmehr um den Ausdruck der oben erwähnten „epileptischen Enzephalopathie“, ist man bewusst dazu übergegangen, für diese EEG-Muster die Bezeichnung GPD („generalized periodic discharges“) zu verwenden und nicht mehr von „generalized periodic epileptiform discharges (GPED)“ oder gar „subtle status epilepticus“ zu sprechen [52]. Diese Bezeichnung betont, dass die Enzephalopathie im Vordergrund steht und sich GPD in der Regel therapeutisch auch nicht durch Antikonvulsiva beeinflussen lassen. Auch Burst-Suppression-Muster können im EEG in dieser Situation auftreten. Zur Verlaufskontrolle können serielle EEGs oder auch ein kontinuierliches EEG-Monitoring sinnvoll sein. Selten lassen sich typische Anfallsmuster im EEG ableiten, wodurch dann ein nonkonvulsiver SE nahegelegt wird und eine aggressive antikonvulsive Therapie indiziert sein kann. Ohne diese Anfallsmuster kann nicht von einem klassischen SE ausgegangen werden, weshalb eine aggressive medikamentöse SE-Therapie nicht zwingend indiziert ist. In solch einer Situation kann es aber durchaus sinnvoll sein, z. B. posthypoxische Myoklonien zur Entlastung des Pflegepersonals und der Angehörigen oder aufgrund einer von ihnen ausgehenden Beeinträchtigung der mechanischen Beatmung z. B. mit Levetiracetam, Clonazepam oder Valproat zu behandeln. In seltenen Fällen wurde sogar eine Muskelrelaxation eingesetzt. Der Einsatz von Anästhetika wie Propofol und Thiopental ist Ausnahmen vorbehalten, in denen z. B. ein SE doch nicht vollkommen ausgeschlossen erscheint. Überlebende Patienten können in den folgenden Wochen ein Lance-Adams-Syndrom mit bewegungsinduzierten Spätmyoklonien entwickeln [67].

### 4.3 Toxische und metabolische Enzephalopathie

Ähnlich wie bei der hypoxischen Enzephalopathie stehen auch bei toxischen oder metabolischen Enzephalopathien, wie der urämischen, septischen oder hepatischen Enzephalopathie, v. a. Vigilanzminderung und Koma im Vordergrund. Myoklonien können vorkommen, aber auch Tremor und Asterixis, was Anlass zur Verwechslung mit epileptischen Anfällen sein kann. In der Abgrenzung zum SE ist das EEG auch hier hilfreich. Es gelten die gleichen Grundsätze und Beobachtungen wie oben bei der hypoxischen Enzephalopathie aufgeführt. Bei der Diagnose dieser Enzephalopathien kann die Bestimmung einiger Laborparameter hilfreich sein. So sind im Rahmen einer urämischen und septischen Enzephalopathie die Serumkonzentrationen von Harnstoff und Stickstoff erhöht [31]. Die hepatische Enzephalopathie kann durch einen Anstieg des Bilirubins oder des Serumammoniaks gekennzeichnet sein.

### 4.4 Tetanus

Das durch *Clostridium tetani* produzierte Toxin Tetanospasmin hemmt die Freisetzung der inhibitorischen Transmitter GABA und Glycin im Rückenmark und führt u. a. zu Störungen der Motorik. Das Vollbild der Erkrankung umfasst eine generalisierte Tonuserhöhung (inkl. Trismus und Ophisthotonus), die unter Umständen mit einem generalisierten epileptischen Anfall verwechselt werden kann. Sie wird durch sensible und akustische Reize provoziert.

## 5 Therapie nach Stufen

### 5.1 Prähospitalphase

Bei einem gesicherten SE oder dringendem klinischem Verdacht auf einen SE wird durch Laien oder den Rettungsdienst ein Benzodiazepin als First-Line-Medikament verabreicht. Laienhelfer sollten bei Verdacht auf Vorliegen eines SE immer den Rettungsdienst verständigen und zwar unabhängig davon, ob sie ein Notfallmedikament verabreichen oder nicht.

Durch den Rettungsdienst bzw. Notarzt muss eine medikamentöse Erstbehandlung erfolgen und der Patient am besten in eine neurologische Klinik transportiert werden, um eine erforderliche Eskalationstherapie sicherzustellen. Die beste Evidenz liegt für die intravenöse Gabe von Lorazepam vor, welches in Metaanalysen der Gabe von Diazepam leicht überlegen war [1, 96]. Allerdings ist die i.v. Applikation und somit die schnelle Therapie durch die Anlage eines peripheren Venenkatheters im Notfall oft verzögert, sodass Benzodiazepine auch intramuskulär, bukkal, rektal, intranasal (z. B. via Nasenzerstäuber oder Nasenspray; [8, 60, 86, 107, 137]) oder im Ausnahmefall auch intraossär verabreicht werden können.

Eine Studie zu i.n. Midazolam in der initialen Therapie des meist nonkonvulsiven SE unter EEG-Kontrolle zeigte eine Responserate von 57 % bei einer medianen Dosis von 5 mg. Der SE wurde im EEG nach durchschnittlich 5:05 min durchbrochen [60]. Eine Metaanalyse konnte nachweisen, dass die alternativen, nicht intravenösen Anwendungsformen sicherer und schneller appliziert werden konnten und nicht mehr Nebenwirkungen aufwiesen [2, 4]. Nach der i.m. Applikation war die i.n. Applikation die wirksamste [4]. Insbesondere für Laien (z. B. Angehörige) oder Rettungspersonal ohne Berechtigung zur i.m. Gabe (Notfallsanitäter) stellt die intranasale Applikation von Midazolam eine in der Notfallsituation einfach zu handhabende Alternative zur i.v. und i.m. Gabe dar. Da sich eine Verzögerung der initialen Benzodiazepingabe von wenigen Minuten negativ auf die Durchbruchsrate des SE auszuwirken scheint [112], sollte der Versuch eines i.v. Zugangs durch den Rettungsdienst auf wenige Minuten beschränkt bleiben und bei Erfolglosigkeit dann rasch auf eine alternative Applikationsform des Benzodiazepins ausgewichen werden. Ein Dosierspray zur intranasalen Applikation kann durch Apotheken hergestellt werden [60] und die i.v. Lösung ist auch zur i.m und i.n. Applikation zugelassen.

#### 5.1.1 Erweiterte prähospitale Maßnahmen und Therapie

Der Schutz vor Selbstgefährdung und das Freihalten der Atemwege muss gewährleistet werden. Festhalten und Beißkeil sind unnütz und verletzungsträchtig.

Maßnahmen durch den Rettungsdienst bzw. Notarzt:

- Sicherstellung der Vitalparameter (ABCDE-Schema)
- Kopf vor Verletzung schützen
- Antikonvulsive Therapie (siehe unten)
- Wenn möglich, Legen mindestens eines stabilen, anfallsungefährdeten (d. h. außerhalb der Ellenbeuge lokalisierten) i.v. Zugangs, ggf. Gabe von 0,9%iger NaCl-Lösung
- Pulsoxymetrie, Blutdrucküberwachung, EKG
- Gabe von Thiamin 100 mg i.v. bei V. a. alkoholassoziierten SGTKA
- Gabe von Glukose 40 % i.v. bei V. a. oder nachgewiesener Hypoglykämie; bei V. a. ethanolassoziierten SE Glukosegabe erst nach Thiamingabe
- O_2_-Insufflation bei O_2_-Sättigung <95 % (via Maske, ggf. Intubation und Beatmung) und symptomatische Temperatursenkung bei Körpertemperatur über 37,5 ^°^C
- Da eine Intubationsbereitschaft immer gesichert sein muss, muss im Zielkrankenhaus eine Intensivüberwachung erfolgen. Zudem besteht die Gefahr einer systemischen Azidose infolge wiederholter motorischer Entäußerungen mit dem Risiko einer Rhabdomyolyse mit sekundärem Nierenversagen.

### 5.2 Therapie der Stufe 1

Die Notwendigkeit einer möglichst frühen Therapie des Status wird durch tierexperimentelle [77] und klinische Daten [1, 19, 62, 74] untermauert.

Benzodiazepine sind die Therapie der 1. Wahl:

- Intravenöses Lorazepam ist die aktuell empfohlene, evidenzbasierte Initialtherapie durch den Rettungsdienst/Notarzt (0,1 mg/kg, max. 4 mg/Bolusgabe, ggf. nach 5 min 1‑mal wiederholen; [1, 23, 46, 95]; EG1A). Allerdings ist auf vielen Rettungsfahrzeugen Lorazepam aufgrund der zu kühlenden Substanz nicht verfügbar.

ODER

- Midazolam intramuskulär oder intranasal (10 mg für >40 kg, 5 mg für 13–40 kg, Einzelgabe; EG1B) oder 0,2 mg/kg i.v., max. 10 mg/Bolusgabe, ggf. nach 5 min 1‑mal wiederholen

ODER

- Clonazepam 0,015 mg/kg (langsame [0,5–1 ml/min] intravenöse Injektion von max. 1 mg, ggf. 1‑mal wiederholen)

ODER

- i.v. Diazepam (0,15–0,2 mg/kg/Gabe, max. 10 mg/Gabe, ggf. nach 5 min 1‑mal wiederholen)

Wenn keine der oben genannten Optionen verfügbar/möglich ist:

- i.v. Phenobarbital (15–20 mg/kg/Gabe, Einzelgabe)

ODER

- Diazepam rektal (0,2–0,5 mg/kg, max. 20 mg/Gabe, Einzelgabe)

Zusammenfassend zeigen Studien, dass bei Erwachsenen intramuskuläres Midazolam, i.v. Lorazepam und i.v. Diazepam etablierte und effektive initiale Therapieoptionen beim SE sind (Level A; [1, 11, 110, 112]). Intramuskuläres Midazolam zeigte zudem eine signifikant effektivere Wirkung bei erwachsenen Patienten mit SE als die i.v. Lorazepamgabe [111, 112]. Bezüglich der nicht i.v. gegebenen Benzodiazepine spricht eine rezente Metaanalyse dafür, dass i.m. Midazolam, gefolgt von i.n. Midazolam, die beiden effektivsten nicht intravenös verabreichten Benzodiazepinapplikationen sind [4]. Eine zu geringe Dosierung des initialen Benzodiazepins und die Gabe von anderen i.v. AED (meist Levetiracetam) waren mit einer niedrigeren Durchbrechungswahrscheinlichkeit von konvulsiven und nonkonvulsiven SE assoziiert [62].

### 5.3 Therapie der Stufe 2

Bei Unwirksamkeit einer adäquaten Dosis des initial gegebenen Benzodiazepins innerhalb der letzten 30 min und fakultativ auch zur Sicherung des Therapieerfolgs bei durchbrochenem SE folgt die Stufe 2 der medikamentösen Therapie:

Seit Ende 2019 besteht die beste Evidenz in dieser Situation für die Gabe von Levetiracetam (60 mg/kg, max. 4500 mg), Fosphenytoin (FPHT, 20 mg/kg, max. 1500 mg) und Valproat (40 mg/kg, max. 3000 mg). Kapur und Mitarbeiter haben in einer prospektiven randomisierten Studie bei Patienten im Alter ab 2 Jahren im generalisierten tonisch-klonischen Status diese i.v. Therapien prospektiv verglichen. Eingeschlossen wurden nur Patienten, die innerhalb der letzten 30 min mindestens 10 mg Diazepam (bei >32 kg 0,3 mg/kg), 4 mg Lorazepam (bei >32 kg 0,1 mg/kg) oder 10 mg Midazolam (bei >32 kg 0,2 mg/kg i.v. bzw. 0,3 mg/kg i.m.) erhalten hatten (Kapur et al. [59], EG1B). Da Fosphenytoin in Deutschland und Österreich zwar zugelassen ist, aber nicht vermarktet wird, und in der Schweiz nicht zugelassen ist, kommen als Medikamente der 1. Wahl praktisch derzeit nur Valproinsäure und Levetiracetam in den genannten Dosierungen infrage:

- Valproat 20 mg/kg, max. 10 mg/kg/min, ggf. nach 10 min wiederholen, kumulativ max. 3000 mg. Für die Weiterbehandlung sollte ein Valproatspiegel von 100–120 µg/ml angestrebt werden.

ODER

- Levetiracetam 30 mg/kg i.v., max. 500 mg/min, ggf. nach 10 min wiederholen, kumulativ max. 4500 mg. Bezüglich der Weiterbehandlung ist derzeit unklar, welcher Spiegel anzustreben ist.

Beide Medikamente sind peripher gut verträglich und können über einen sicher intravenös liegenden peripheren Zugang gegeben werden. Bei Valproinsäure besteht eine Kontraindikation für Patienten mit Mitochondropathie. Ferner ist zu beachten, dass es bei gleichzeitiger Gabe von Carbapenemen (z. B. Meropenem) oft nicht gelingt, einen ausreichenden Wirkspiegel aufzubauen [9].

Im Jahr 2019 wurden 2 Komparatorstudien publiziert, die Phenytoin (PHT, 20 mg/kg über 20 min infundiert) mit Levetiracetam (LEV, 40 mg/kg über 5 min infundiert) bei Kindern und Jugendlichen mit einem konvulsiven SE verglichen [25, 76]. Beide Studien fanden keine signifikanten Unterschiede in der Effektivität oder Verträglichkeit. Der Status sistierte in 70 % (LEV) vs. 64 % (PHT; [76]) bzw. 50 % (LEV) vs. 60 % (PHT) der Fälle [25]. Eine in 2020 publizierte Metaanalyse zum Vergleich von LEV mit anderen i.v. AED kam zu dem Schluss, dass LEV bezüglich der Wirksamkeit gegenüber PHT, VPA und Lorazepam nicht unterlegen war und dass LEV insgesamt das beste Verträglichkeitsprofil aufwies [20].

Wenn LEV oder VPA kontraindiziert oder nicht verfügbar sind, können auch die anderen zugelassenen i.v. verfügbaren AED gegeben werden:

- PHT-Infusionskonzentrat 20 mg/kg i.v. (max. 50 mg/min und über einen separaten i.v. Zugang). Zu beachten: Die akute hochdosierte i.v. Phenytoingabe sollte immer unter Intensivüberwachung mit Monitoring von Blutdruck und EKG erfolgen. Keine Phenytoingabe über Magensonde (mangelnde Resorption) oder intramuskulär (gewebetoxisch!). Der Sicherheit (Stabilität) des i.v. Zugangs kommt bei PHT wie auch bei Thiopental wegen der Gefahr von Gewebenekrosen bei Extravasaten besondere Bedeutung zu. Für die Weiterbehandlung sollte ein Phenytoinspiegel von 20–25 µg/ml angestrebt werden.
- Alternativ oder bei Kontraindikation gegenüber PHT oder bei Unwirksamkeit von frühzeitig verabreichtem PHT steht Phenobarbital (PB) zur Verfügung. Eine prospektive Vergleichsstudie von PB und VPA sprach für eine Überlegenheit von PB bezüglich der Effektivität (81 % vs. 44 % Kontrollrate und 7 % vs. 31 % Rezidivrate) bei nicht signifikant verschiedener Tolerabilität, und ein aktueller Review kam zu dem Ergebnis, dass PB sogar das Wirksamste der i.v. Medikamente sei, wobei Lacosamid und VPA Verträglichkeitsvorteile aufwiesen [14].
- Phenobarbital 15–20 mg/kg i.v. (max. 100 mg/min, höhere Gesamtdosen sind unter Intensivmonitoring, nach Intubation oder unter Beatmungsbereitschaft möglich). Cave: Interaktionsrisiken und mögliche Intoxikation bei zusätzlicher Verwendung von VPA. Für die Weiterbehandlung sollten Spiegel von 30–50 µg/ml angestrebt werden.
- Ebenfalls als Therapie der 2. Wahl kommt die i.v. Gabe von Lacosamid (LCM) in Betracht. Eindeutige Evidenz für die angemessene Dosis liegt nicht vor. Als häufig eingesetzte initiale Dosis wurden 5 mg/kg als Kurzinfusion über 15 min beschrieben [14], z. T. wird 200 mg/15 min als maximale Infusionsgeschwindigkeit vorgeschlagen. Die akute hochdosierte Gabe von i.v. LCM sollte unter EKG-Monitoring stattfinden.

Eine randomisierte prospektive Studie mit relativ kleiner Fallzahl verglich Wirksamkeit und Tolerabilität von Phenytoin und Lacosamid bei nonkonvulsiven Anfällen (nicht SE) und fand eine vergleichbare Wirksamkeit und Tolerabilität: Anfälle sistierten bei 19 von 30 (63 %) Patienten im LCM-Arm und bei 16 von 32 (50 %) Patienten im PHT-Arm [56]. Es liegen bisher Berichte zu mehr als 500 Anwendungen von LCM beim Status epilepticus, meist als 3. oder weiteres Medikament, vor [118]. Die Wirksamkeit war bei Patienten mit konvulsiven und nonkonvulsiven SE-Formen vergleichbar und bei fokalen motorischen SE am höchsten [118]. Die am häufigsten verwendete Dosierung war 5 mg/kg über ≥15 min [14, 53, 61, 118]. In einer kleineren prospektiven Studie wurde berichtet, dass mit der Gabe von >9 mg/kg LCM therapeutische LCM-Spiegel von 10–20 mg/l erzielt wurden. Es ergab sich jedoch keine Korrelation zwischen Spiegel und Wirksamkeit [93]. LCM ist (wie LEV) nicht zur Therapie des SE zugelassen. Es liegen, wie für einige der anderen Optionen auch, bisher keine prospektiven Studien zur Wirksamkeit und Verträglichkeit beim SE vor. Wegen der möglichen Verlängerung der PQ-Zeit stellt ein AV-Block 2. oder 3. Grades eine Kontraindikation dar. Bei herzkranken Patienten soll der Einsatz nur mit Vorsicht erfolgen. Die Anwendung von LCM i.v. scheint sicher zu sein, wenn die Anwendungsbeschränkungen bezüglich kardialer Nebenwirkungen beachtet werden.

Navarro und Mitarbeiter haben in einer kontrollierten prospektiven Studie untersucht, ob die präklinische gleichzeitige Gabe von Clonazepam und LEV der alleinigen Gabe von Clonazepam überlegen ist. Die Studie erbrachte ein negatives Ergebnis. De facto war die Durchbrechungsrate im Monotherapiearm höher und es war daher statistisch nicht möglich, eine Überlegenheit der Kombination zu zeigen [90]. Für die gleichzeitige Gabe von Benzodiazepinen und eines i.v. verfügbaren AED liegt somit keine ausreichende Evidenz vor.

Spätestens, wenn nach adäquat dosiertem Benzodiazepin und der Gabe einer der hier genannten Substanzen der SGTKA nicht durchbrochen ist, muss beim konvulsiven SE in der Regel eine Weiterbehandlung auf der Intensivtherapiestation erfolgen. Bei nonkonvulsiven SE-Formen ist im Einzelfall nach klinischer Einschätzung zu entscheiden.

### 5.4 Therapie der Stufe 3

Ein Status epilepticus, welcher auf die wiederholte Gabe von Benzodiazepinen und die folgende Therapieeskalation mit intravenöser Gabe eines Zweitlinienantiepileptikums nicht sistiert, wird als therapierefraktärer Status epilepticus (RSE) bezeichnet. Das übergeordnete Ziel in der Therapie des RSE ist es, sowohl die klinische als auch die elektroenzephalographische Anfallsaktivität zu durchbrechen. Dazu ist die intravenöse Verabreichung von Anästhetika notwendig. In diesem Zusammenhang ist meist eine Sicherung der Atemwege mittels Intubation indiziert. Da die Wahrscheinlichkeit eines generalisierten konvulsiven Status epilepticus (GKSE) durch Gabe eines weiteren nicht anästhetischen Antikonvulsivums gering ist, sollten beim generalisierten konvulsiven und nicht konvulsiven RSE rasch anästhetische Antikonvulsiva (d. h. intravenös applizierte Sedativa) eingesetzt werden. Die drei zu diesem Zweck eingesetzten Substanzen sind Midazolam (MDL), Propofol (PRO) und Thiopental (THP); oftmals werden MDL und PRO in Kombination verwendet, während THP meist alleinig und nicht selten erst nach einem Versagen von MDL und PRO eingesetzt wird.

Die Rationale hinter dem entschlossenen Vorgehen besteht darin, sowohl akute systemische als auch chronische neuronale Schädigungen verhindern zu können [130]. Akute systemische Komplikationen wie ein Lungenödem oder – potenziell lebensgefährdende – Herzrhythmusstörungen können sehr früh (innerhalb der ersten 30 min) im Verlauf des generalisierten konvulsiven SE auftreten [138].

Das Evidenzniveau für die Behandlung des RSE mit Anästhetika ist niedrig. In einem systematischen Review ließ sich auf der Basis von retrospektiven Studien zwar ein Vorteil für Barbiturate (THP und das heute nicht mehr verfügbare Pentobarbital [PTB]) im Vergleich mit MDL und PRO bezüglich eines Rückfalls des RSE finden [21]. Diese Ergebnisse können jedoch nicht ohne Weiteres in den klinischen Alltag übertragen oder in Leitlinienempfehlungen übernommen werden. Die Limitation dieser Ergebnisse besteht darin, dass die jeweilige Barbituratdosis EEG-gesteuert bis zur Suppression der Hintergrundaktivität titriert wurde. Dahingegen erfolgte die Dosistitration für PRO und MDL hauptsächlich bis zur klinischen und elektroenzephalographischen SE-Kontrolle, ohne dass hierfür notwendigerweise ein Burst-Suppression-EEG-Muster erreicht wurde. Relevante Nebenwirkungen wie insbesondere eine arterielle Hypotonie waren in der Barbituratgruppe stärker ausgeprägt. Die Mortalität war im Mittel mit 48 % in allen Gruppen gleich hoch.

Eine multizentrische randomisierte einfach-blinde Studie untersuchte die Wirksamkeit von Barbituraten und Propofol (mit Behandlungserfolg in 22 % respektive 43 % ohne signifikanten Unterschied) bei Patienten mit einem auf Benzodiazepine und ein weiteres Antikonvulsivum refraktären SE [100]. Nach drei Jahren konnten nur 24 der notwendigen 150 Patienten rekrutiert werden, was die Schwierigkeiten bei der Durchführung einer klinischen Studie auf diesem Gebiet veranschaulicht. Auch wenn in dieser Studie bei keinem Patienten ein Propofolinfusionssyndrom (PRIS: Herzinsuffizienz, schwere metabolische Azidose, Rhabdomyolyse und Nierenversagen bei einer Behandlung von mehr als 48 h mit mehr als 5 mg/kg/h [135]) auftrat, so wurde dieses in einer retrospektiven Studie bei 14 von 31 mit Propofol behandelten Patienten mit einem RSE, von denen 3 verstarben, beobachtet [57].

In einer retrospektiven Studie mit 33 Patienten wurde MDL mit THP verglichen. Dabei zeigte sich erneut eine höhere Nebenwirkungsrate unter THP und eine kürzere Hospitalisationsdauer unter MDL sowie ein signifikant besseres Outcome nach 6 Monaten [10].

Eine weitere randomisierte Studie untersuchte den Einsatz von PRO versus MDL im RSE mit Erreichen einer Anfallsunterdrückung (keine Burst-Suppression-Aktivität). Bei 23 Patienten erwies sich keines der beiden Therapeutika als signifikant überlegen, wobei PRO zu einer signifikant kürzeren Hospitalisation führte [81]. Ein Cochrane-Database-Review, der PRO mit THP zur Behandlung des RSE verglich, kam ebenfalls zu dem Schluss, dass nach wie vor keine robusten randomisierten kontrollierten Studien mit hilfreicher Evidenz bei der Behandlung des RSE zur Verfügung stehen [94].

Zusammengefasst lassen die vorliegenden Daten bisher keine Empfehlung zugunsten einer der Substanzen THP, MDL oder PRO zu. Unabhängig von ihrem Stellenwert für die Therapie des RSE ist der Einsatz der Barbiturate in der Intensivtherapie allerdings aufgrund ihres ungünstigeren Nebenwirkungsprofils und pharmakokinetischer Nachteile (Immunsuppression, negative Inotropie, längere Eliminationshalbwertszeiten u. a.) in den letzten Jahren deutlich zurückgegangen. Auch die Interimsanalyse eines multinationalen Registers zur Behandlung des RSE zeigte, dass derzeit MDL am häufigsten eingesetzt wird (56 %), gefolgt von PRO (35 %) und Barbituraten (8 %), wobei in Europa PRO häufiger eingesetzt wird als MDL [35]. Die Autoren dieser Leitlinie empfehlen, dass in jedem Zentrum diejenige Substanz zur Behandlung des RSE eingesetzt werden soll, mit der die meiste Erfahrung besteht.

Folgende Therapieschemata werden in Anlehnung an die Richtlinien der NCS [17] nach Versagen der beiden initialen Antikonvulsiva innerhalb 60 min begonnen:

- Midazolam 0,2 mg/kg i.v. als Bolus, Erhaltungsdosis EEG-gesteuert (Ziel: Anfallskontrolle und, falls erreichbar, ein Burst-Suppression-Muster, maximale Dosisrate bis 2,9 mg/kg/h) für 24 h [37]

ODER

- Propofol 2 mg/kg i.v. als Bolus, Erhaltungsdosis EEG-gesteuert (Ziel: Burst-Suppression-Muster, ca. 4–10 mg/kg/h) für 24 h. Cave: Propofolinfusionssyndrom (s. oben)

ODER

- Thiopental 5 mg/kg als Bolus, Erhaltungsdosis EEG-gesteuert (Ziel: Burst-Suppression-Muster, ca. 0,5–5 mg/kg/h) für 24 h. Wegen der negativen Inotropie von Thiopental ist häufig die zeitgleiche Gabe positiv inotroper Substanzen (meist Noradrenalin, seltener Dopaminperfusor) erforderlich.

Bei der Behandlung des RSE gibt es bezüglich der Therapieintensität drei verschiedene Strategien. Die am wenigsten intensive Therapie besteht in der klinischen und EEG-dokumentierten Unterdrückung der Anfallsaktivität. Letzteres setzt allerdings voraus, dass EEG-geschultes Personal die Therapie überwacht und erkennen kann, wann die Anfallsunterdrückung gelungen ist. Die vermutlich am häufigsten gewählte Strategie umfasst die Induktion einer suppressionsdominanten Burst-Suppression-Aktivität (Verhältnis von „Burst“ zu „Suppression“ 1:10–20), da dieses Muster auch von nicht spezifisch im EEG ausgebildeten Behandelnden relativ leicht erkannt werden kann. Tierexperimentelle Daten zeigen allerdings, dass sich das Gehirn unter der Burst-Suppression-Aktivität in einem deutlich übererregbaren, potenziell schädigenden Zustand befindet [66]. Die dritte Möglichkeit besteht in der Induktion einer isoelektrischen EEG-Kurve zur Behandlung des RSE, wozu aber meistens THP eingesetzt werden muss, da eine isoelektrische Kurve mit MDL kaum je zu erreichen ist und mit PRO hohe Dosen verabreicht werden müssen, mit denen ein potenziell erhöhtes Risiko der Entwicklung eines PRIS besteht. Diese Strategie mag einerseits das Gehirn effektiv beruhigen und eine allfällig begleitende Hirnschwellung bekämpfen, ist jedoch mit den substanziellen Nachteilen und Nebenwirkungen der Barbiturattherapie sowie weit längerer Intensivstationsverweilzeit und Hospitalisationsdauer verbunden.

Gemäß einer neueren retrospektiven Kohortenstudie kann zur Drittlinienbehandlung des RSE auch eine kürzere tiefere Narkose mit Erreichen einer Burst-Suppression im Gegensatz zu einer oberflächlicheren Narkose zur Anfallsdurchbrechung (ohne Erreichen einer Burst-Suppression) erwogen werden [87]. Allerdings bleibt die Evidenz auch für diese Behandlungsstrategie schwach und weiterführende Untersuchungen stehen aus.

Ebenso zeigte eine kürzlich veröffentlichte retrospektive Kohortenstudie, dass eine frühzeitige (innerhalb von 48 h nach Symptombeginn) Behandlung des RSE mit kontinuierlichen intravenösen Anästhetika signifikant mit einer guten Prognose, weniger häufiger mit einer Einleitung einer Burst-Suppression im EEG sowie mit einem geringeren Risiko des Bedarfs einer THP-Verabreichung verbunden war [78].

Vier retrospektive Kohortenstudien zur Drittlinienbehandlung mit Anästhetika (mit kumulativ *n* > 800) haben gezeigt, dass die Induktion eines iatrogenen Komas zur RSE-Behandlung mit einer erhöhten Mortalität assoziiert ist [65, 80, 126, 128, 129]. Neben noch unklaren Gründen für diese Assoziation spielen möglicherweise anästhetikaassoziierte Komplikationen wie Infektionen und schwere arterielle Hypotension, die die Gabe von Vasopressoren erfordern, eine gewisse Rolle. Dabei legt eine dieser Studien nahe, dass besonders bei Patienten im fokalen nicht konvulsiven RSE auf die Gabe von Anästhetika verzichtet werden kann [80], und eine weitere Studie beschreibt eine Assoziation zwischen der kontinuierlichen Gabe von Anästhetika und erhöhter Mortalität nur bei Patienten, bei denen sich ein SE außerhalb des Krankenhauses entwickelt [129].

Die Richtlinien der europäischen Intensivmedizinischen Gesellschaft [22] wie auch die der amerikanischen Critical Care Society empfehlen die kontinuierliche EEG-Ableitung bei der Therapieeinleitung, -überwachung und -ausleitung eines RSE [17].

Untersuchungen, die sich mit der Frage beschäftigen, wie rasch die Anästhetika nach erfolgreich behandeltem RSE wieder ausgeschlichen werden sollen, bestehen weiterhin keine. Hier handelt jedes Zentrum nach seiner Erfahrung. Erste Schritte, mittels Maschinenlernen Algorithmen und Muster zu identifizieren, die die Frage beantworten könnten, wann und wie ein solches Ausschleichen gesteuert werden könnte, sind eingeleitet [102].

Die publizierten Erfahrungen zur Anwendung des Bispectral-Index(BIS)-Monitoring beim SE zeigen zwar wie in anderen Indikationsstellungen eine ausreichende Steuerung der Narkosetiefe [88], sie können jedoch nicht die kontinuierliche EEG-Dauerableitung – insbesondere in der Phase der Reduktion von Anästhetika – ersetzen.

#### Empfehlung

Zusammenfassend.

- Der refraktäre Status epilepticus soll mit Propofol oder Midazolam oder einer Kombination der beiden oder mit Thiopental in anästhetischen Dosen so rasch als möglich (<48 h nach Symptombeginn) behandelt werden (Empfehlungsstärke: stark).

#### Empfehlung

- Die Therapie des refraktären konvulsiven Status epilepticus soll auf einer Intensivstation bei intubierten Patienten erfolgen (Empfehlungsstärke: stark).
- Die Therapieeinleitung, deren Überwachung und das Ausschleichen der Anästhetika sollten mittels kontinuierlichen EEG-Monitorings erfolgen (Empfehlungsstärke: Empfehlung).
- Als Therapieziel bei der Einleitung eines therapeutischen Komas können folgende EEG-Muster erwogen werden: a) reine Anfallsunterdrückung, b) suppressionsdominante Burst-Suppression-Aktivität, c) isoelektrische EEG-Kurve (Empfehlungsstärke: offen).
- Es kann beim fokalen nicht konvulsiven refraktären Status epilepticus auf die Einleitung eines therapeutischen Komas verzichtet werden (Empfehlungsstärke: offen).

### 5.5 Therapie der Stufe 4, Management des superrefraktären Status epilepticus

#### Einführung

Ein superrefraktärer Status epilepticus (SRSE) liegt vor, wenn die klinische und/oder elektroenzephalographische Anfallsaktivität nach Einleitung einer Therapie mit kontinuierlich applizierten Anästhetika anhält oder zeitnah nach initial erfolgreicher Therapie wieder auftritt, dies beinhaltet auch die Reduktion und/oder das Absetzen der Anästhetika [109]. Die in Stufe 3 eingesetzten Anästhetika umfassen Substanzen, die die Inhibition am GABA-Rezeptor verstärken, dies sind Midazolam, Propofol und Thiopental. Im Folgenden werden Empfehlungen für therapeutische Ansätze beim SRSE ausgesprochen. Die Evidenz für deren Wirksamkeit ist sehr niedrig, sie beruht fast ausschließlich auf kleinen, meist retrospektiven Fallserien und Kasuistiken. Neben einem potenziellen Bias hinsichtlich der Publikation von erfolgreich verlaufenen therapeutischen Interventionen sind der mögliche Spontanverlauf des SRSE und die antiepileptische Komedikation zu berücksichtigen.

### Pharmakologische Interventionen

#### Barbiturate

Auch nach Versagen von kontinuierlich intravenös applizierten Anästhetika kann der Einsatz von hochdosierten Barbituraten beim SRSE erfolgreich sein. In einer retrospektiven Studie wurden 31 Patienten beschrieben, bei denen nach einer medianen Dauer des SE von 6,5 Tagen intravenös Pentobarbital (Metabolit von Thiopental, in den USA, aber nicht in Deutschland, Österreich und der Schweiz zugelassen) in einer Dosis von 0,5–3,7 mg/kg/h appliziert wurde [97]. Nach 6 Tagen eines Burst-Suppression-Musters im EEG war der SE bei 90 % der Patienten durchbrochen. Mit Absetzen des Pentobarbitals traten bei 15 der 31 Patienten wieder Anfälle auf, bei 12 dieser 15 Patienten konnten diese durch intravenöses Phenobarbital kontrolliert werden. Unerwünschte Effekte umfassten Pneumonie (32 %), Harnwegsinfekte (13 %), tiefe Beinvenenthrombose und Ileus (je 10 %).

##### Empfehlung

Zusammenfassend kann bei Patienten mit SRSE der Einsatz von hochdosierten Barbituraten unter Abwägung von Nutzen und Risiken erwogen werden. Da Pentobarbital in Deutschland, Österreich und der Schweiz nicht zugelassen ist, kann hier der chemische Ausgangsstoff Thiopental zum Einsatz kommen (Empfehlungsstärke: offen).

#### Ketamin

Ketamin ist das einzige zugelassene Anästhetikum, welches seine Wirkung beim SE über eine Blockade des exzitatorischen NMDA-Rezeptors entfaltet.

In drei retrospektiven Studien mit 135 Patienten mit SRSE (Ausschluss von posthypoxischen Enzephalopathien) konnte Ketamin diesen bei 15 von 53 Patienten (28,3 %; [43]), bei 18 von 28 Patienten (64,3 %; [53]) und bei 52 von 54 Patienten (96,3 %; [103]) durchbrechen. Bei der letztgenannten Studie kann ggf. die Koadministration von Propofol die hohe Wirksamkeit erklären. Aber auch unter ausschließlicher Berücksichtigung der beiden erstgenannten Studien lag die Erfolgsquote bei über 40 %. Ein klinisch relevanter Vorteil von Ketamin ist das Fehlen einer kardiopulmonalen Depression, somit kann in der Regel auf den Einsatz von Katecholaminen verzichtet werden.

##### Empfehlung

Zusammenfassend kann bei Patienten mit SRSE der Einsatz von intravenös appliziertem Ketamin erwogen werden. Die Koadministration einer GABAergen Substanz kann erfolgen, um möglichen neurotoxischen Effekten des NMDA-Rezeptor-Antagonisten entgegenzuwirken (Empfehlungsstärke: offen).

#### Inhalationsanästhetika

In einer systematischen Übersichtsarbeit (13 Studien mit 28 erwachsenen Patienten) konnte gezeigt werden, dass der SRSE in 93 % der Fälle durch Inhalationsanästhetika durchbrochen werden konnte [139]. In vielen Fällen kam es jedoch bei Beendigung der Therapie zu einem Wiederauftreten von Anfällen. Bei fast allen Patienten wurde Isofluran verwendet, welches mit GABA-, Glutamat- und Glyzinrezeptoren interferiert, zudem hemmt es aktivierte Kaliumkanäle. Der einzige relevante unerwünschte Effekt ist eine arterielle Hypotension.

##### Empfehlung

Zusammenfassend kann bei Patienten mit SRSE der Einsatz von inhalativem Isofluran erwogen werden. Vor dem Hintergrund der technischen Herausforderungen einer mehrtägigen Gabe einer volatilen Substanz und der allgemein geringen klinischen Erfahrung kann Isofluran (nur bei ausgewählten Fällen) zum Einsatz kommen (Empfehlungsstärke: offen).

#### Enterale Applikation „klassischer“ Antiepileptika

Es liegt eine Reihe von Berichten zum Einsatz von klassischen Antiepileptika, die nicht in intravenöser Form erhältlich sind, beim SE vor. In den meisten Fallserien werden Daten von Patienten mit refraktärem SE und mit SRSE gemischt.

*Perampanel* ist ein Antagonist am AMPA-Rezeptor. In einer systematischen Übersichtsarbeit wurden 10 Arbeiten mit 69 Episoden eines SE (bei 68 Patienten) analysiert [16]. Vor Einsatz von Perampanel bestand der SE schon für 9 h bis 35 Tage. Unter einer Dosis von 2–32 mg wurde der SE nach 1 h bis 4 Wochen in 17–100 % der Fälle durchbrochen. Die Heterogenität der Ergebnisse deutet auf die schwierige Interpretierbarkeit dieser Daten hin. In einer retrospektiven Studie aus vier europäischen Ländern konnte Perampanel den SRSE bei 6 von 23 Patienten (26 %) unterbrechen [119].

*Topiramat* ist ein Antiepileptikum mit mehreren Wirkmechanismen, unter anderem wirkt es antagonistisch am exzitatorischen AMPA-Rezeptor. Der Einsatz beim SE wurde in vier Studien mit 35 Patienten beschrieben [13]. 6 Patienten hatten einen SRSE, bei 5 Patienten wurde Topiramat als letzte Substanz appliziert, bei 4 dieser Patienten wurde die Anfallsaktivität dadurch beendet. In einer Studie aus Frankfurt und Marburg wurden 40 Patienten mit SRSE mit Topiramat behandelt, bei 8 Patienten (20 %) konnte der SE durchbrochen werden [34].

Für weitere Antiepileptika, wie *Oxcarbazepin* und *Pregabalin*, liegen sehr kleine retrospektive Fallserien vor, deren Aussagekraft hinsichtlich der Wirksamkeit beim SRSE äußerst limitiert ist. Daher werden diese hier nicht explizit aufgeführt.

##### Empfehlung

Zusammenfassend kann bei Patienten mit SRSE der Einsatz von enteralen „klassischen“ Antiepileptika erwogen werden. Die größten Fallserien liegen für Perampanel und Topiramat vor, beide Substanzen haben einen antagonistischen Effekt am AMPA-Rezeptor. Da der Effekt mitunter erst nach Tagen bzw. Wochen auftritt, sind ein spontanes Sistieren der Anfallsaktivität und/oder der Effekt anderer zwischenzeitlich eingesetzter Substanzen nicht auszuschließen (Empfehlungsstärke: offen).

#### Allopregnanolon

Spezifische Neurosteroide modulieren allosterisch die Aktivität am GABA-A-Rezeptor und erhöhen dadurch dessen Sensitivität gegenüber GABAerg wirkenden Substanzen. Allopregnanolon, ein natürlicher Metabolit von Progesteron, steigert allosterisch die Leitfähigkeit am GABA-A-Rezeptor. Obwohl die Daten aus einer initialen Phase-I/II-Studie beim SRSE vielversprechend waren, war Allopregnanolon in einer randomisierten doppelblinden placebokontrollierten Studie hinsichtlich des primären Endpunkts (anhaltende Anfallsfreiheit nach Absetzen des Anästhetikums) nicht besser als die Kontrollgruppe (44 % vs. 42 %; *p* = 0,88; [104]).

##### Empfehlung

Zusammenfassend soll bei Patienten mit SRSE Allopregnanolon, nach dem negativen Ergebnis einer randomisierten kontrollierten Studie, nicht eingesetzt werden (Empfehlungsstärke: stark).

### Nicht pharmakologische Interventionen

#### Ketogene Diät

Die ketogene Diät (KD) ist eine strikte kohlenhydratarme Ernährung, welche zu erhöhten freien Fetten führt, dies resultiert in der Produktion von Ketonkörpern, welche die Blut-Hirn-Schranke passieren. Der exakte antiepileptische Wirkmechanismus ist nicht geklärt, aber letztlich führen die Ketonkörper zu einer neuronalen Hyperpolarisation und somit zu einer reduzierten neuronalen Exzitabilität.

In einer retrospektiven Studie erhielten 10 Patienten mit SRSE (vorherige mediane Dauer des SE 22 Tage) eine KD [132]. Bei 9 Patienten wurde eine Ketose erreicht, bei all diesen Patienten konnte die Anfallsaktivität nach medianen 3 Tagen durchbrochen werden. 3 Patienten wiesen eine transiente Azidose und eine Hypertriglyzeridämie auf, es gab keine weiteren unerwünschten Effekte.

Diese vielversprechenden Ergebnisse führten zu einer multizentrischen Phase-I/II-Studie hinsichtlich der Wirksamkeit von KD bei erwachsenen Patienten mit SRSE [18]. 15 Patienten erhielten KD über die Magensonde, die mediane Dauer des SE vor KD betrug 10 Tage. Die Ketose wurde bei allen Patienten innerhalb von 2 Tagen erreicht. Bei 11 von 14 Patienten, bei denen die KD über mehrere Tage durchgeführt werden konnte, ließ sich der SRSE nach 1 bis 10 Tagen durchbrechen. Unerwünschte Effekte umfassten metabolische Azidose, Hyperlipidämie, Konstipation, Hypoglykämie, Hyponatriämie und Gewichtsverlust.

##### Empfehlung

Zusammenfassend sollte bei Patienten mit SRSE die ketogene Diät zum Einsatz kommen. Die Gabe über die Magensonde ist im Alltag von Intensivstationen gut umsetzbar, die Therapie ist komplikationsarm, Daten aus einer retrospektiven, aber auch aus einer prospektiven Phase-I/II-Studie zeigen die gute Wirksamkeit (Empfehlungsstärke: Empfehlung).

#### Hypothermie

Der antiepileptische Effekt einer reduzierten Körperkerntemperatur beruht wahrscheinlich auf mehreren Mechanismen, diese umfassen eine präsynaptische Alteration mit reduzierter Ausschüttung von exzitatorischen Transmittern, eine Änderung von postsynaptischen spannungsabhängigen Kanälen und Einflüsse auf Membraneigenschaften und Ionenpumpen.

Eine systematische Übersichtsarbeit konnte 13 Studien mit 40 Patienten mit einem „schwer behandelbaren“ SE identifizieren, die mit Hypothermie behandelt worden sind [139]. Die Körpertemperatur wurde für mediane 48 h auf 33 ^°^C gesenkt. Bei 25 der 40 Patienten (62,5 %) konnte die Anfallsaktivität durchbrochen werden. Auch bei diesem überraschend positiven Ergebnis muss ein Publikationsbias berücksichtigt werden.

In einer randomisierten kontrollierten Studie wurde bei 268 Patienten mit einem generalisierten konvulsiven SE untersucht, bei welchem Anteil der Patienten die systemische Kühlung auf 33 ^°^C für 24 h mit nachfolgender Wiedererwärmung über 24 h zu einem fehlenden funktionellen Defizit 90 Tage später führt [68]. Hinsichtlich dieses primären Endpunkts konnte kein Unterschied zwischen den Gruppen festgestellt werden. Einer der sekundären Endpunkte war die Entwicklung hin zu einem im EEG nachgewiesenen SE, dies zeigte sich in der hypothermiebehandelten Gruppe signifikant seltener. Der Anteil der Patienten, die einen refraktären SE oder einen SRSE entwickelt haben, wurde durch die Hypothermie nicht beeinflusst. Diese Studie gibt einen Hinweis auf einen antiepileptischen Effekt der Hypothermie in der frühen Phase des SE, die Daten zeigen jedoch nicht an, dass die Senkung der Körpertemperatur einen Effekt beim SRSE hat.

##### Empfehlung

Zusammenfassend soll bei Patienten mit SRSE die systemische Hypothermie mit dem Therapieziel Anfallsunterbrechung und Besserung der funktionellen Prognose auf Basis der aktuellen Datenlage nicht zum Einsatz kommen (Empfehlungsstärke: stark).

#### Epilepsiechirurgie

Bei ausgesuchten Patienten mit pharmakoresistenter fokaler Epilepsie stellt die resektive Epilepsiechirurgie den erfolgreichsten Therapieansatz dar. Falls sich der SRSE auf einen eindeutigen Anfallsfokus, idealerweise auf Basis einer epileptogenen Läsion, zurückführen lässt, kann die Resektion der epileptogenen Zone die Anfallsaktivität ggf. beenden. Eine retrospektive Studie hat 9 Patienten mit SRSE gezeigt, bei denen 10 bis 54 Tage nach Beginn des SE mithilfe der Elektrokortikographie der Anfallsfokus identifiziert und letztlich reseziert wurde [6]. Dies führte bei 5 der 8 Patienten zu Anfallsfreiheit.

In einer systematischen Übersichtsarbeit wurde die Wirksamkeit der Vagusnervstimulation (VNS) mit akuter Implantation bei 38 Patienten mit refraktärem SE und SRSE untersucht [29]. Bei 28 Patienten (74 %) war die akute VNS mit einer Beendigung der Anfallsaktivität assoziiert. Die mediane Dauer des SE vor Implantation betrug 18 Tage, die bis zur Beendigung des SE nach Implantation 8 Tage, es gab jeweils eine erhebliche Spanne. Auch bei diesen Daten ist ein erheblicher Publikationsbias zu berücksichtigen. Für den Einsatz der tiefen Hirnstimulation in der Therapie des SE liegt keine ausreichende Evidenz vor.

##### Empfehlung

Zusammenfassend können bei Patienten mit SRSE die resektive Epilepsiechirurgie und die Vagusnervstimulation erwogen werden. Gerade der resektive Ansatz hängt von der Identifikation der epileptogenen Zone und idealerweise von der Möglichkeit ab, diese entfernen zu können, ohne ein persistierendes neurologisches Defizit auszulösen (Empfehlungsstärke: offen).

#### Elektrokonvulsive Therapie

Der Einsatz der elektrokonvulsiven Therapie beim schwer behandelbaren Status epilepticus wurde kasuistisch beschrieben, auch hier ist ein erheblicher Publikationsbias zu berücksichtigen.

In einer Übersichtsarbeit wurden 14 Publikationen mit 19 Patienten (15 Erwachsene und 4 Kinder/Jugendliche) zusammengefasst, die elektrokonvulsive Therapie kam sowohl beim refraktären als auch beim SRSE zum Einsatz [140]. Eine Reduktion der Anfallslast wurde bei 4 (21 %) und ein Sistieren der Anfallsaktivität bei 7 (37 %) der 19 Patienten beobachtet. Die Dauer der Anfallskontrolle war recht heterogen, sie lag überwiegend bei 2 Wochen bis 3 Monaten. Somit kam es also in der Regel zu einem Wiederauftreten der Anfallsaktivität.

##### Empfehlung

Zusammenfassend kann die elektrokonvulsive Therapie beim SRSE in Einzelfällen erwogen werden (Empfehlungsstärke: offen).

## 6 Versorgungskoordination

In die Versorgung sind Personen, die statusgefährdete Patienten in Einrichtungen oder zu Hause betreuen, Rettungsstellen, Rettungs- und Notfallsanitäter, Notärzte, Notaufnahmen, Intermediate-Care- und Intensivstationen und deren Personal involviert. Kinder werden üblicherweise in Kinderkliniken mit Notaufnahme betreut. Hier besteht eine Leitlinie der GNP.

Die aktuelle Studie zum Status generalisierter tonisch-klonischer Anfälle [59] und die rezente Leitlinie der AES zum SE generalisierter tonisch-klonischer Anfälle [46] machen bezüglich der Therapie dieser Statusform bis auf Dosisanpassungen keine Unterschiede mehr im Management von Kindern ab 2 Jahren bis zum Hochbetagten. Allerdings ist zu beachten, dass im Kindesalter andere Epilepsiesyndrome im Vordergrund stehen und verschiedene Statusformen mit anderer Häufigkeit auftreten als bei Erwachsenen. Daher ist die Betreuung durch erfahrene Neuropädiater und pädiatrische Intensivmediziner erforderlich. Dies gilt besonders für Kinder im Alter unter 2 Jahren, welche sich auch bezüglich Pharmakokinetik und -dynamik erheblich von Erwachsenen unterscheiden.

## 7 Mortalität und Erste-Hilfe-Maßnahmen

Erste-Hilfe-Maßnahmen wurden unter 5.1 Prähospitalphase behandelt.

Die Mortalität eines SE liegt zwischen 3 und 39 % [72]. Bei erwachsenen Patienten mit einem konvulsiven SE liegt sie im Durchschnitt bei 15,9 % [91]. Sie hängt stark vom Alter, der Ätiologie und der Schwere und Dauer eines SE ab. Auch die Therapie spielt eine Rolle, denn insbesondere für die späte und insuffiziente Gabe von Benzodiazepinen scheint ein Einfluss auf die Durchbrechungsraten und auch auf die Mortalität möglich, wenn nicht sogar wahrscheinlich [62, 63, 73, 91, 136]. In den letzten Jahren wurden mehrere klinische Scores entwickelt, welche eine prognostische Einschätzung schon früh im Verlauf ermöglichen, beispielhaft sollen hier der Status Epilepticus Severity Score (STESS-Score) und der Epidemiology-based Mortality Score in Status Epilepticus (EMSE-Score) erwähnt werden [45, 70, 99].

## 8 Medizinethische Aspekte mit Therapierelevanz

Die Behandlung des RSE und SRSE stellt insbesondere bei relevant vorerkrankten Patienten des höheren Lebensalters sowie bei Patienten mit einer fortgeschrittenen malignen Grunderkrankung mit, aber auch ohne eine rechtlich bindende Patientenverfügung eine besondere medizinethische Herausforderung dar. Da die Stufe 3 der Therapie des nonkonvulsiven SE (NCSE) den Beginn intensivmedizinischer Maßnahmen bedeutet, ist der diesbezügliche Patientenwille zu berücksichtigen, wobei eine schriftliche Patientenverfügung hierfür die Grundlage darstellt. Weil diese häufig allgemein formuliert ist, sollte obligat mit oder ohne vorliegende Patientenverfügung im Gespräch mit den Angehörigen bzw. Bezugspersonen der individuelle und rezente Patientenwille eruiert werden. Von besonderer klinischer Relevanz ist dies für den NCSE mit quantitativer Bewusstseinsminderung, da dieser zum einen intrahospital gegenüber dem CSE zahlenmäßig überwiegt. Zu beachten ist ferner, dass das Vorliegen eines Komas beim NCSE einen ungünstigen Prädiktor darstellt [131]. Beim CSE andererseits fehlen in der durch Zeitnot geprägten Akutsituation häufiger Ätiologie und Prognosemarker und Angehörige können teils noch nicht einbezogen werden.

Vergleichende Studien zu Mortalität, Morbidität und Länge der Krankenhausbehandlung zur Therapie des NCSE mit quantitativer Bewusstseinsminderung, mit oder ohne Therapie der Stufe 3 für den relevant vorerkrankten Patienten des höheren Lebensalters liegen ebenso wenig vor wie für Patienten mit einer fortgeschrittenen malignen Grunderkrankung. Es bestehen jedoch Hinweise, dass für beide Gruppen eine erhöhte Mortalität bzw. ein schlechteres neurologisches Outcome anzunehmen sind [78, 84, 124].

Es gilt daher, vor dem Hintergrund u. a. der Ätiologie, des Alters, der Vorerkrankungen und etwaiger aufgetretener, die Prognose verschlechternder Akutkomplikationen, wie z. B. einer Pneumonie, die Prognose und Dauer einer intensivmedizinischen Therapie und die Wahrscheinlichkeit der Notwendigkeit invasiver Maßnahmen im Verlauf (z. B. Tracheotomie) individuell einzuschätzen und vor diesem Hintergrund dem mutmaßlichen Patientenwillen gerecht zu werden, ob im Vorfeld verschriftlicht oder nicht. In Bezug auf Patienten mit maligner Grunderkrankung gilt es zudem, die Vereinbarkeit einer zeitnah notwendigen onkologischen Therapie mit der geplanten Statusbehandlung sowie der hierfür erforderlichen Verweildauer auf der Intensivstation zu berücksichtigen, wobei insbesondere abgeschätzt werden sollte, ob und wann der für eine weitere onkologische Therapie erforderliche Karnofsky-Index erreicht werden kann.

### Empfehlung

- Bei Verzicht auf Therapiemaßnahmen der Stufe 3 kann eine Fortführung der Therapie auf Stufe 2 oder die Einleitung einer palliativen Behandlung folgen (Empfehlungsstärke: offen).
- Bei Einleitung bzw. Fortführung oder Eskalation einer intensivmedizinischen Therapie bei Patienten mit RSE und SRSE sollten der Patientenwille und das Vorliegen einer Patientenverfügung beachtet werden (Empfehlungsstärke: Empfehlung)

## Abkürzungen

AED: Antiepileptikum
CSE: konvulsiver Status epilepticus
DGfE: Deutsche Gesellschaft für Epileptologie
DGKN: Deutsche Gesellschaft für Klinische Neurophysiologie und funktionelle Bildgebung
DGN: Deutsche Gesellschaft für Neurologie
E: Empfehlungsstärke
EG: Evidenzgrad
GKSE: generalisierter konvulsiver Status epilepticus
GTKSE: generalisierter tonisch-klonischer Status epilepticus
ILAE: International League Against Epilepsy
KI: Kurzinfusion
KS: Konsensstärke
LCM: Lacosamid
LEV: Levetiracetam
NCSE: nonkonvulsiver Status epilepticus
ÖGfE: Österreichische Gesellschaft für Epileptologie
ÖGN: Österreichische Gesellschaft für Neurologie
PB: Phenobarbital
PHT: Phenytoin
RSE: refraktärer Status epilepticus
PTB: Pentobarbital
SE: Status epilepticus
SGN: Schweizer Gesellschaft für Neurologie
SGTKA: Status generalisierter tonisch-klonischer Anfälle
SLgE: Schweizer Liga gegen Epilepsie
SRSE: superrefraktärer Status epilepticus
THP: Thiopental
VPA: Valproat
